# Microplastic sources, formation, toxicity and remediation: a review

**DOI:** 10.1007/s10311-023-01593-3

**Published:** 2023-04-04

**Authors:** Ahmed I. Osman, Mohamed Hosny, Abdelazeem S. Eltaweil, Sara Omar, Ahmed M. Elgarahy, Mohamed Farghali, Pow-Seng Yap, Yuan-Seng Wu, Saraswathi Nagandran, Kalaivani Batumalaie, Subash C. B. Gopinath, Oliver Dean John, Mahendran Sekar, Trideep Saikia, Puvanan Karunanithi, Mohd Hayrie Mohd Hatta, Kolajo Adedamola Akinyede

**Affiliations:** 1 School of Chemistry and Chemical Engineering, David Keir Building, Queen’s University Belfast, Stranmillis Road, Belfast, BT9 5AG Northern Ireland, UK; 2 Green Technology Group, Environmental Sciences Department, Faculty of Science, Alexandria University, Alexandria, 21511 Egypt; 3 Chemistry Department, Faculty of Science, Alexandria University, Alexandria, Egypt; 4 Environmental Science Department, Faculty of Science, Port Said University, Port Said, Egypt; 5 Egyptian Propylene and Polypropylene Company (EPPC), Port-Said, Egypt; 6 Department of Agricultural Engineering and Socio-Economics, Kobe University, Kobe, 657-8501 Japan; 7 Department of Animal and Poultry Hygiene & Environmental Sanitation, Faculty of Veterinary Medicine, Assiut University, Assiut, 71526 Egypt; 8 Department of Civil Engineering, Xi’an Jiaotong-Liverpool University, Suzhou, 215123 China; 9 Centre for Virus and Vaccine Research, School of Medical and Life Sciences, Sunway University, 47500 Subang Jaya, Selangor Malaysia; 10 Department of Biological Sciences, School of Medical and Life Sciences, Sunway University, 47500 Subang Jaya, Selangor Malaysia; 11 Department of Biomedical Sciences, Faculty of Health Sciences, Asia Metropolitan University, 81750 Johor Bahru, Malaysia; 12 Faculty of Chemical Engineering & Technology, Universiti Malaysia Perlis (UniMAP), 02600 Arau, Perlis Malaysia; 13 Institute of Nano Electronic Engineering, Universiti Malaysia Perlis (UniMAP), 01000 Kangar, Perlis, Malaysia; 14 Micro System Technology, Centre of Excellence, Universiti Malaysia Perlis (UniMAP), Pauh Campus, 02600 Arau, Perlis Malaysia; 15 Faculty of Science and Natural Resources, Universiti Malaysia Sabah, 88400 Kota Kinabalu, Sabah Malaysia; 16 Faculty of Pharmacy and Health Sciences, Royal College of Medicine Perak, Universiti Kuala Lumpur, 30450 Ipoh, Perak Malaysia; 17 Girijananda Chowdhury Institute of Pharmaceutical Science, Guwahati Assam, India; 18 Department of Anatomy, Faculty of Medicine, Manipal University College Malaysia (MUCM), Melaka, Malaysia; 19 Department of Pharmacology, Faculty of Medicine, University of Malaya, Kuala Lumpur, Malaysia; 20 Centre for Research and Development, Asia Metropolitan University, 81750 Johor Bahru, Johor Malaysia; 21 Department of Medical Bioscience, University of the Western Cape, Bellville, Cape Town, 7530 South Africa; 22 Biochemistry Unit, Department of Science Technology, The Federal Polytechnic, P.M.B.5351, Ado Ekiti, 360231 Ekiti State Nigeria

**Keywords:** Microplastic pollution, Water treatment, Biodegradable plastics, Microplastic detection, Microplastic control, Microplastic toxicity

## Abstract

Microplastic pollution is becoming a major issue for human health due to the recent discovery of microplastics in most ecosystems. Here, we review the sources, formation, occurrence, toxicity and remediation methods of microplastics. We distinguish ocean-based and land-based sources of microplastics. Microplastics have been found in biological samples such as faeces, sputum, saliva, blood and placenta. Cancer, intestinal, pulmonary, cardiovascular, infectious and inflammatory diseases are induced or mediated by microplastics. Microplastic exposure during pregnancy and maternal period is also discussed. Remediation methods include coagulation, membrane bioreactors, sand filtration, adsorption, photocatalytic degradation, electrocoagulation and magnetic separation. Control strategies comprise reducing plastic usage, behavioural change, and using biodegradable plastics. Global plastic production has risen dramatically over the past 70 years to reach 359 million tonnes. China is the world's top producer, contributing 17.5% to global production, while Turkey generates the most plastic waste in the Mediterranean region, at 144 tonnes per day. Microplastics comprise 75% of marine waste, with land-based sources responsible for 80–90% of pollution, while ocean-based sources account for only 10–20%. Microplastics induce toxic effects on humans and animals, such as cytotoxicity, immune response, oxidative stress, barrier attributes, and genotoxicity, even at minimal dosages of 10 μg/mL. Ingestion of microplastics by marine animals results in alterations in gastrointestinal tract physiology, immune system depression, oxidative stress, cytotoxicity, differential gene expression, and growth inhibition. Furthermore, bioaccumulation of microplastics in the tissues of aquatic organisms can have adverse effects on the aquatic ecosystem, with potential transmission of microplastics to humans and birds. Changing individual behaviours and governmental actions, such as implementing bans, taxes, or pricing on plastic carrier bags, has significantly reduced plastic consumption to 8–85% in various countries worldwide. The microplastic minimisation approach follows an upside-down pyramid, starting with prevention, followed by reducing, reusing, recycling, recovering, and ending with disposal as the least preferable option.

## Introduction

Water is an essential resource on the surface of the earth, crucial for all industrial, agricultural, and humans activities as well as the biological processes of all non-human beings, to sustain life (Eltaweil et al. 2022; Hosny et al. 2022a; El-Maghrabi et al. 2021; Crini and Lichtfouse 2019). Although water covers more than two-thirds of the earth’s surface, only 0.1% is available for fresh water to all living organisms, including humans. Despite the actual availability of fresh water resources, humans are dramatically disrupting the natural ecosystems and contaminating this water by dumping vast amounts of various types of water contaminants, including organic such as pharmaceutical wastes, dyes, plastics, and pesticides, and inorganic wastes, e.g. heavy metals, into different aquatic bodies (Hosny et al., 2022b; Mahmoud et al. 2022; Abd El-Monaem et al. 2022; Rashid et al. 2021; Osman et al. 2022; Naqash et al. 2020). Consequently, these contaminants and their remediation started to gain the researcher’s interest by investigating numerous water treatment techniques (Abdelfatah et al. 2021; Oliveira et al., 2020). One of the emerging contaminants that seriously affect water quality is microplastics, which are thoroughly discussed in this review article.

Microplastics, which are tiny plastic particles measuring less than 5 mm in length, have been found to have significant negative impacts on both human health and the environment. The term "microplastics" was first coined 19 years ago by Thompson et al. (2004), who studied oceanic plastic pollution in the UK. Since then, microplastics have attracted the attention of the scientific community, governments, non-governmental organisations, and others. While plastics are relatively new materials that came into use during the second half of the last century (Gündoğdu and Çevik 2017), their excessive production and use in various products and industries have resulted in a significant threat to the environment (Osman et al. 2020; Qasim et al. 2020). Primary microplastics, such as cosmetic microbeads used in facial washes, are intentionally made tiny and are therefore classified as such (Wang et al. 2019). Nanoplastics are of particular concern as they pose a greater risk to living organisms than microplastics due to their higher abundance and reactivity. Their small size allows them to easily penetrate living cells and reach remote locations, exacerbating their potential harm (Sharma et al. 2022).

**Fig. 1:**
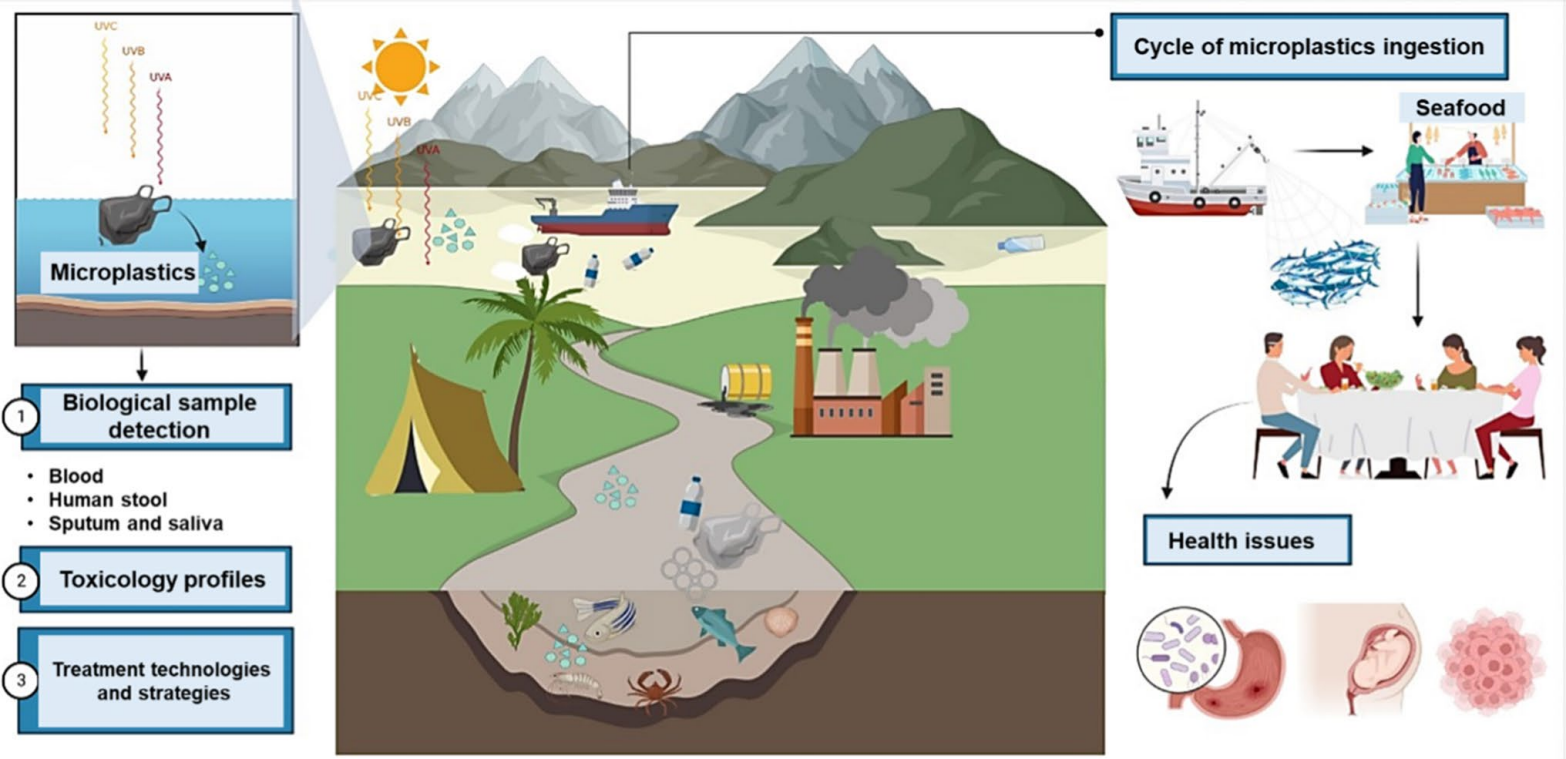
Microplastic effects and pathways on the environment and human health. Microplastics' formation is detectable in several biological samples. Microplastic has toxicological effects, necessitating the implementation of treatment technologies. The cycle of microplastic ingestion ends primarily in seafood and its associated health problems. UVA, UVB, and UVC are different ultraviolet (UV) radiation types. UVA has the longest wavelength, is the least energetic, and is the most common type of UV radiation. UVB has a medium-range wavelength and is more energetic than UVA. UVC has the shortest wavelength and is the most active type of UV radiation

This review focuses on various aspects of microplastics, including their formation, biological detection, toxicological profile, detrimental health effects, and potential treatments, as shown in Fig. 1. Further, this article includes sources and effects of microplastics on the environment and human health, global initiatives and responses to reduce the release of microplastics, public perception and awareness of microplastics, and various approaches that can be taken to improve this. The review also examines the link between microplastic pollution, climate change, and biodiversity loss. It compares potential treatment techniques and control strategies to mitigate microplastic pollution and enhance the reuse and recycling of plastics.

## Production of plastic and microplastics

During the last 70 years, global plastic production has risen from 1.5 million tonnes to approximately 359.0 million tonnes (Bui et al., 2020) and is expected to reach 500.0 million tonnes by 2025 (Huang et al., 2021a). In 2013, China produced approximately 63.0 million tonnes of plastic, accounting for most plastic production worldwide. When this number is combined with the plastic production of other Asian countries, the total plastic production reaches approximately 114.0 million tonnes. (Ryan 2015). The European Union was the second-largest region for plastic production, with nearly 50.0 million tonnes produced. North America also contributed significantly, with 49.0 million tonnes of plastic produced. However, Latin America, Commonwealth countries, Africa, and the Middle East collectively produced only 37.0 million tonnes of plastic.

Unfortunately, the majority of plastic waste is being incinerated, dumped in landfills, and released into the environment, causing significant environmental and health problems (Wang et al. 2020a), with only a tiny percentage that does not exceed 10.0% recycled in the USA (Cessi et al. 2014). In addition, it is worth mentioning that plastic wastes constitute more than 75.0% of marine waste materials, owing to their rigid and non-biodegradable nature (Zhang et al., 2021a). Although the Mediterranean Sea region is considered one of the essential resources for human life, it has unfortunately become one of the most highly polluted areas with plastics and microplastics (Cózar et al. 2015). The majority of plastics released into the Mediterranean are contributed by five countries, with Turkey being the largest contributor of approximately 144.0 tonnes per day of plastic waste, followed by Spain at 126 tonnes, Italy at 90.0 tonnes, Egypt at 77.0 tonnes, and France at 66.0 tonnes (Sharma et al., 2021).

**Fig. 2:**
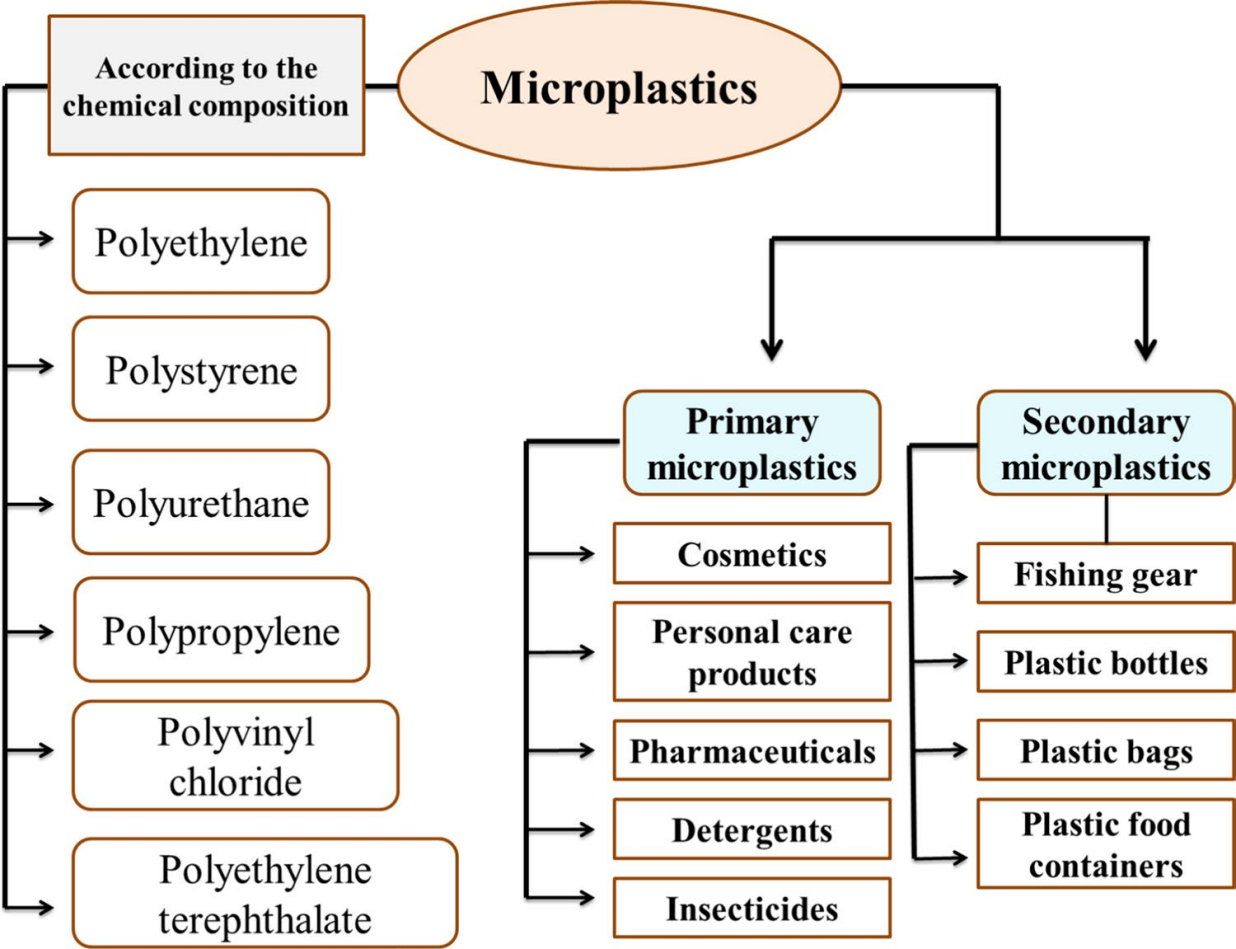
Different classifications of microplastics. Microplastics can be classified into two categories: primary microplastics and secondary microplastics. Primary microplastics are intentionally manufactured and added to consumer and commercial products like cosmetics, personal care products, pharmaceuticals, detergents, and insecticides. Secondary microplastics, on the other hand, are unintentionally formed by the breakdown of larger plastic materials through physical, chemical, or biological processes, such as fishing gear, plastic bottles, plastic bags, and plastic food containers. Microplastics can also be classified based on their chemical composition, which includes polyethylene, polypropylene, polystyrene, and other materials

Furthermore, microplastics can also form unintentionally through the degradation of larger polymers, which can occur due to physical, chemical, or biological factors, such as tire debris. These microplastics are known as secondary microplastics, as depicted in Fig. 2 (Andrady 2017). On the other hand, primary microplastics are intentionally added to consumer and commercial products, such as cosmetics, detergents, paints, medications, nappies, and insecticides (Duis and Coors 2016). Microplastics can be categorised into five major types: fragments, fibres, foam, pellets, and films (Anderson et al. 2017). Moreover, microplastics can be classified into six categories based on their chemical composition: polyethylene, polystyrene, polypropylene, polyurethane, polyvinyl chloride, and polyethylene terephthalate, as shown in Fig. 2 (He et al. 2022).

In recent years, the production of microplastics has significantly risen, with their concentrations detected on the coasts of some marine areas reaching thousands of particles per cubic meter. Without adequate measures, these numbers are expected to double in the next few years (Isobe et al. 2019). Moreover, the issue is further complicated by the lack of reliable and accurate sampling techniques, which means that the reported concentrations of microplastics in marine ecosystems may not reflect the actual amounts, leading to a potential underestimation of the problem (Brandon et al. 2020).

**Fig. 3:**
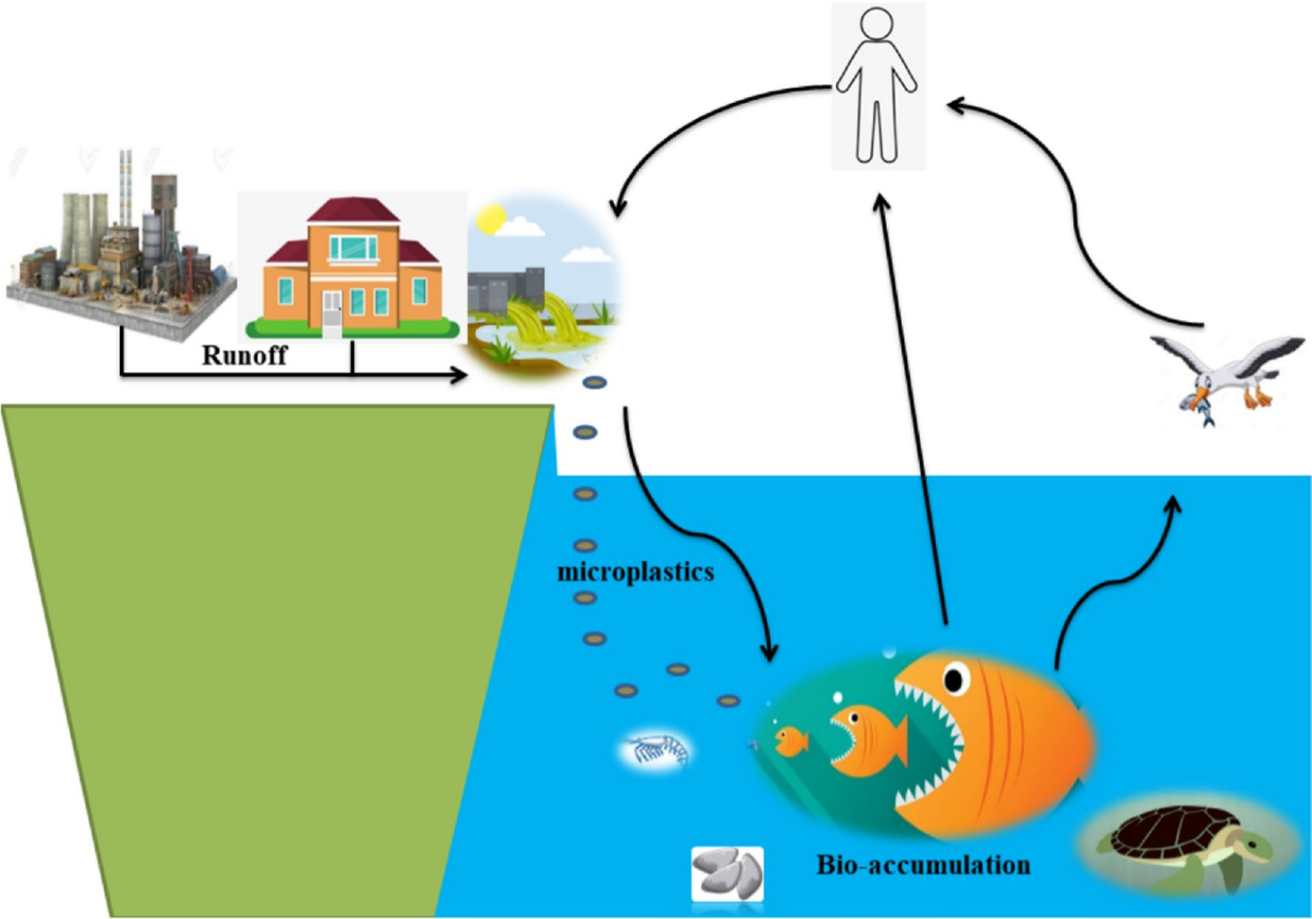
Life cycle of microplastics in the environment. The discharge resulting from diverse activities flows into aquatic systems, introducing microplastics into the food chain and their subsequent bioaccumulation in the tissues of aquatic organisms. This accumulation can result in significant adverse effects on the aquatic ecosystem, and these effects can be directly transmitted to humans and birds

These tiny particles significantly impact the environment, particularly aquatic bodies, as they can accumulate and leach toxic organic and inorganic pollutants, such as persistent organic pollutants and heavy metals (Van Emmerik et al. 2018). Microplastics are also known for their stability and inability to degrade, meaning they can persist in the environment for decades (Xiang et al. 2022). The life cycle of microplastics, which involves bioaccumulation, is shown in Fig. 3. This cycle usually begins with the release of primary or secondary microplastics into the terrestrial and aquatic ecosystems, followed by their transport into water systems.

Consequently, microplastics enter the food chain of aquatic organisms and undergo bioaccumulation in their tissues, gradually working their way up the trophic levels as zooplankton, small fish, larger fish, and other organisms consume them. Swallowing these pollutants has been shown to have toxic effects on aquatic life, including fish, oysters, mussels, and sea turtles, such as compromising their immune and digestive systems and potentially leading to their demise (Matsuguma et al. 2017; Hipfner et al. 2018; Caron et al. 2018). Microplastics have the potential to directly affect human health, as they can enter the human food chain through the consumption of contaminated fish or other aquatic organisms. Studies have shown that microplastics can have cytotoxic effects on human brain cells (Schirinzi et al. 2017). In addition to carrying toxic chemicals, microplastics can adsorb various contaminants, including antibiotics, due to their large surface area, further exacerbating the problem of microplastic pollution (Li et al. 2018) . Furthermore, the cycle of microplastics in the environment continues as they may be excreted by humans or discharged as plastic waste materials.

Microplastics have been recently monitored in drinking water in many countries and in bottles of mineral water (Schymanski et al. 2018). Hence, it is imperative to develop new methods and innovative techniques for removing plastics from water sources, as conventional methods are ineffective in eliminating microplastics due to their small size. This has led to an increase in the prevalence and persistence of microplastics in the environment. (Hou et al. 2021). The review thoroughly investigates several innovative treatment strategies, including the removal of plastic microbeads from cosmetics and personal care products, the utilisation of bioplastics like polyhydroxyalkanoates that can be biologically degraded in the environment, the enhanced reuse and recycling of plastics, the development of efficient waste separation strategies in waste treatment facilities, and the use of bioremediation treatments (Wu et al. 2017; Calero et al. 2021).

It is worth noting that research into removing microplastics is relatively new, having only started in 2014. The number of publications related to microplastic removal was very low in the first two years, with only one publication each in 2014 and 2015. However, this number has significantly increased recently, reaching 145 in 2020. This increase in research could be attributed to a combination of factors, including the free time researchers had due to coronavirus disease 2019 (COVID-19) lockdowns and a growing scientific interest in addressing the microplastics issue and finding effective solutions in line with global initiatives to minimise plastic waste.

### Sources of microplastics and problem statement 

There is ample evidence that watercourses contain microplastics with various shapes, sizes, densities, structures, and chemical compositions (Auta et al. 2017). Table 1 lists various types of microplastics in different countries, with numerous forms and sizes.

**Table 1:** Sources, chemical composition, shape, size, and the location position of the main commonly used microplastics

Source	Composition and structure			Location	Reference
	Chemical composition	Shape	Size		
Shower gels	Polyethylene	Irregular shapes	422 ± 185 μm	Beijing, China supermarkets	Lei et al. (2017)
Facial cleansers	Polyethylene	Spherical and irregular shapes	Higher than 0.5 mm	New Zealand supermarkets	Fendall and Sewell (2009)
Car tyres	Polypropylene/acrylic/nylon/rubber	Fragment/fibre	Higher than 500 μm	Queensland's Gold Coast	Ziajahromi et al. (2020)
Beverage products	Polyamide/acrylonitrile–butadiene–styrene/poly(ester-amide)/poly(ethylene terephthalate)	Fibres/fragments	0.1–3 mm	Supermarket (Walmart) of Mexico City, Mexico	Zhou et al. (2021)
Facial scrubs	Polyethylene/polyvinyl chloride	Spherical/irregular/granular	85 to 186 μm	Mainland China	Cheung and Fok (2017)
Textile industrial area	Polyester	Fibre	0.1–1 mm	Shaoxing city, China	Deng et al. (2020a)
Cosmetic products	Polyethylene	Irregular/granular/spherical	54–115 μm	United Arab Emirates	Habib et al. (2020)
Plastic mulch	Polyester, polypropylene	Fibre/fragment/foam/film	Higher than 500 μm	Qinghai-Tibet plateau, west of China	Feng et al. (2021)
Industrial sources	Polyethylene/nylon/polypropylene	Films/fragments/lines/granules/sheets/lines	0.5–1.0 mm	Northwestern Pacific Ocean	Hou et al. (2021)
Mariculture activities	Polyester/polypropylene/polyethylene/polyamide (nylon)/polystyrene/polyoxymethylene/polyetherurethane/polybutylene terephthalate	Fragments/flakes/ fibre/foam	Less than 0.25 mm	Maowei Sea, China	Anderson et al. (2017)
Fishing and shipping activities	Ionomer surlyn/acrylic (acryl fibre)/polyetherimide/polyphenylene sulphide/ethylene vinyl alcohol/acrylonitrile/nylon/polyisoprene/polyvinyl chloride/ethylene–vinyl acetate/polyurethane	Fibre/pellet/fragment	1489 ± 1017 μm	Port Blair Bay, Andaman Islands	He et al. (2022)
Anthropogenic activity	Polystyrene/polyethylene/polypropylene	Fibre/styrofoam/fragment/film/pellet	Less than 0.5 mm	Three Gorges Reservoir, China	Bui et al. (2020)
Personal care products/facial cleansers/sewage sludge	Polystyrene/polyester/amino thermoset plastic/polyallyl di glycol carbonate	Fragment/pellet/foam/film/line	0.355–0.999 mm	The Laurentian Great Lakes of the USA	Huang et al. (2021a)
Urban sewage	Polyethylene/polystyrene/polypropylene	Fragment/lines/ foam/film	1–4.75 mm	The Southern Caspian Sea Coasts	Ryan (2015)
Industrial areas	Polyester/nylon	Fibre/foam/ Fragment	50 μm to 2000 μm	Ciwalengke River, Indonesia	Wang et al. (2020a)
Fishery activities and human domestic sewage/ building industry	Polyvinylchloride/polyethylene/polyamide	Fibres/pellets/films/fragments	less than 0.5 mm	Nanxun Reef in Nansha Islands, South China Sea	Zhang et al. (2021a)
Urbanisation	Polyethylene/polypropylene	Pellets/fragments/film/line/foam	0.3–4.75 mm	Southwest coast of India	Cózar et al. (2015)
Industrial area	Polyethylene/polypropylene/nylon	Fibres/fragments	0.1–5 mm	Northern shores of the United Arab Emirates	Sharma et al. (2021)
Industrial activities	Polyethylene/polyethylene terephthalate/polyester/poly(vinyl stearate)/polypropylene/cellulose	Fragment/fibre/pellet	1001–2000 mm	The Karasu River Erzurum, Turkey	Brandon et al. (2020)
Tertiary industry	Polyethylene/polypropylene/polyacrylonitrile/polyethylene terephthalate	Fragment/fibre/film	500 μm to 5 mm	Tourist city in China	Van Emmerik et al. (2018)
Sludge and wastewater treatment plants	Polyamide (i.e. nylon)/polyethylene/polypropylene	Fragment/fibre/film/granule	0.003–0.05 mm	The Persian Gulf	Xiang et al. (2022)
Anthropogenic activity	polypropylene/polyethylene terephthalate/polyamide(nylon)/polystyrene/polyethylene	Fibre/film/pellet/granular	Less than 2 mm	Wuhan, China	Matsuguma et al., (2017); Hipfner et al. (2018); Caron et al. (2018)
Local inputs/ocean transport	Polypropylene/polyester/polyester/polyethylene	Fibre/flake/film/granule	2.0–2.5 mm	Antarctic seawater	Schymanski et al. (2018)
Artificial ecosystems	Polyethylene/rayon/polypropylene	Fibre/flake/film/granule	Less than1 mm	Southwestern China	Čulin and Bielić, (2016)
Domestic, agriculture effluent, industry, upstream inflow, and airborne settlement	Polyethylene terephthalate/polyethylene/polypropylene/polystyrene/polycarbonate/polyvinyl chloride/cellulose propionate/polyamide/ethylene–vinyl acetate copolymer	Pellets/fragments	0.05–5 mm	Xiangjiang river, China	Alomar et al. (2016)
Plastic industries	polypropylene/polyester/nylon/polystyrene	Fibre/line/spherule/fragment/granule/film	Less than 0.5 mm	South Yellow Sea, China	Rochman (2018)
Commercial fish species	Polyethylene terephthalate/polyethylene/polypropylene/polyamide/phthalocyanine	Fibres/fragments	Higher than 215 μm	Seri Kembangan, Malaysia	Karbalaei et al. (2019)
Anthropogenic activities	Polyethylene terephthalate/cellulose acetate/ polyvinyl chloride/polypropylene/polyethylene	Fibres/spheres/fragments	Higher than or equal to 1 to less than10 μm,	Drinking water treatment plants, the Úhlava River (Czech Republic)	Naji et al. (2017)

The size and shape differ greatly depending on the microplastic source and type.

**Fig. 4:**
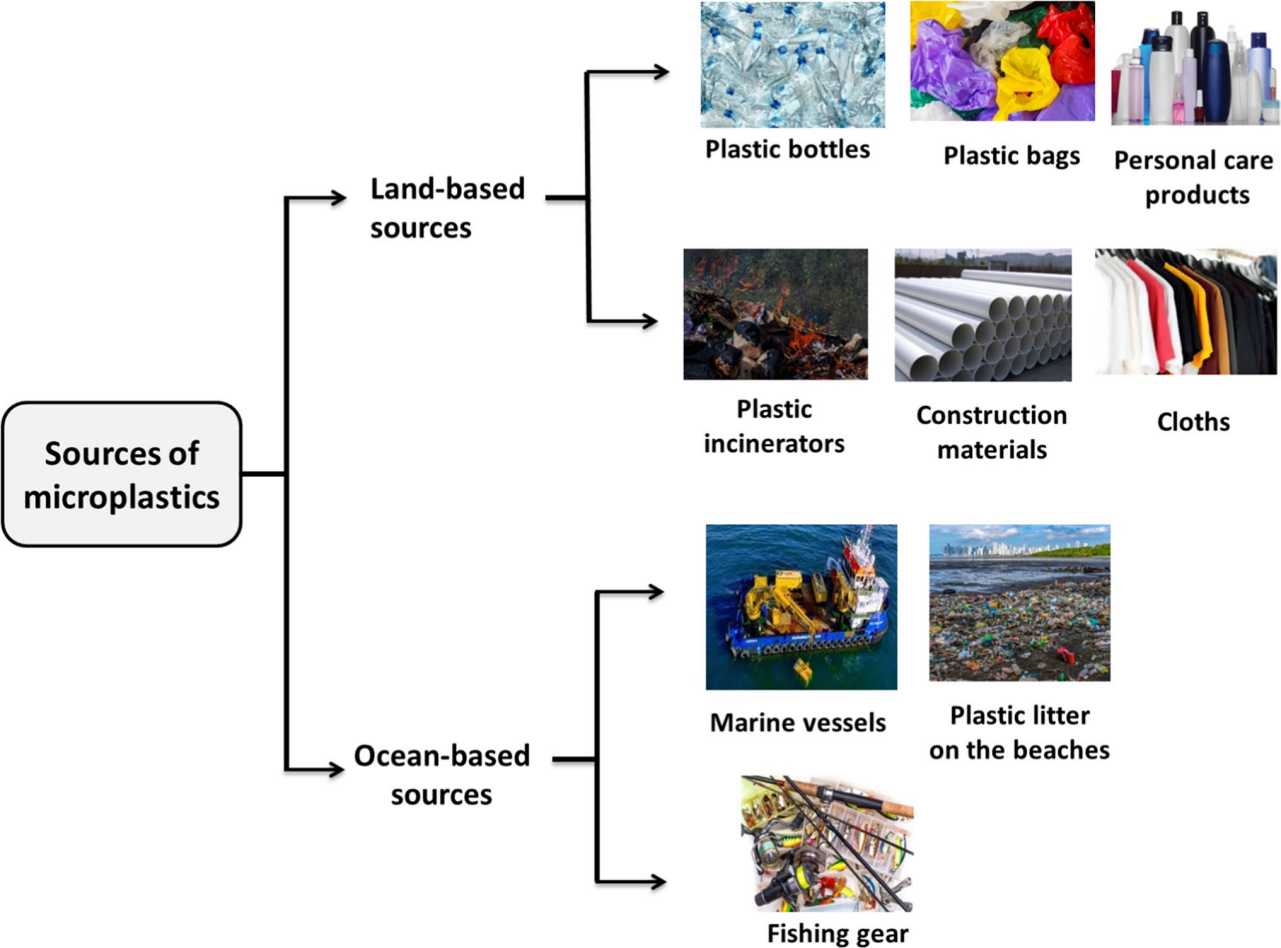
Land-based and ocean-based microplastics' sources. Land-based sources contribute 80–90% of microplastics to water bodies, which include plastic bags, plastic bottles, personal care products, plastic incinerators, construction materials, and textiles. Ocean-based sources contribute 10–20% of microplastic discharge into water bodies, mainly marine vessels, plastic litter on beaches, and fishing gear

Generally, there are many sources of microplastics, but they are mainly classified into land- and ocean-based sources, as shown in Fig. 4.

#### Land-based sources of microplastics

Land-based sources are responsible for 80–90% of microplastics in water bodies (Duis and Coors 2016). These sources include plastic bags, bottles, personal care products, construction materials, and clothing. Plastic incinerators, which generate bottom ash that contains microplastics, are also a land-based source of these particles (Yang et al. 2021). Construction materials, household products, packaging items, food and drink packaging waste, and waste generated from shipbuilding are some of the most significant sources of larger plastic objects on land (Čulin and Bielić, 2016; Alomar et al. 2016). Sewage sludge and industrial activities, particularly those using granules and small resin pellets, are other probable sources of microplastic discharge into the aquatic environment (Rolsky et al. 2020; Hale et al. 2020). In addition to medicines and construction materials, certain cosmetics and personal care products are also considered potential sources of plastic pollution, as they may contain microplastics used as drug carriers or as ingredients (Rochman 2018). Face washes, hand soaps, hand gels, laundry detergents, washing powder, toothpaste, facial creams, mascaras, lipsticks, sunblock, and shower gels are some of the common examples of such products (Guerranti et al. 2019). Many synthetic fibres, such as polyester, nylon, and acrylics, have been found to shed off clothing and discharge with the stream wastewater into water bodies (Carney Almroth et al. 2018). Tire wear and tear of cars greatly release microplastics into the environment (Kole et al. 2017). Therefore, It is clear that numerous sources of microplastics must be effectively controlled and minimised to the greatest extent possible.

Single-use products made of polymeric plastics, such as drinking bottles, straws, cutlery, coffee cups, and bags, have been identified as a significant source of plastic pollution in the environment (Fadare et al., 2020). Furthermore, the excessive use of single-use face masks made of plastic polymers, such as polyesters and polypropylenes, during the coronavirus disease 2019 (COVID-19) has significantly increased microplastic waste (Fadare and Okoffo 2020). Replacement of conventional plastic materials used in face masks and other products with sustainable, eco-friendly materials that can be easily degraded is necessary should future waves of COVID-19 occur.

#### Ocean-based sources of microplastics

Approximately 10–20% of microplastics discharged into the aquatic environment come from ocean-based sources, including seaside tourism, commercial fishing, marine vessels, and offshore industries (Li 2018; Karbalaei et al. 2019). Discarded or lost fishing gear, such as plastic monofilament lines and nylon nets, are a significant source of microplastics that can float at different depths in the ocean (Naji et al. 2017). Over 600,000 tonnes of fishing gear are thrown away in the ocean each year, contributing to the problem (Good et al. 2010). Shipping microplastic waste, commonly released from shipping and naval vessels, also adds to the problem (Peng et al. 2018). Moreover, a massive quantity of plastic waste from offshore industries, such as petrochemicals, is being released into marine ecosystems (Calero et al., 2021). While the contribution of ocean-based sources to microplastic pollution is not as high as land-based sources, it is still significant. Control strategies are needed to reduce this contribution.

## Microplastics pollution problem and international response 

Recently, microplastics have been found in freshwater ecosystems, including rivers, lakes, estuaries, wetlands, and groundwater (Wong et al., 2020; Du et al., 2021). While the concentration of microplastics in freshwater environments is lower than in marine environments, contamination of freshwater is rapidly increasing at an unprecedented rate (Li et al. 2020a). Water quality, human activities, urbanisation, and wastewater treatment technologies are key factors that regulate microplastic pollution levels in freshwater systems (Zhang et al. 2022a). Wetlands are among the largest ecosystems that receive microplastics from municipal, agricultural, and industrial wastewater, making them a significant sink for microplastics (Kumar et al. 2021). Microplastics are more likely to settle in lakes than rivers as they represent a closed-water body and have lower current rates that control microplastic transport (Lu et al. 2021a).

Additionally, the presence of microplastics in freshwater is influenced by varying rainfall patterns (Eo et al. 2019). With the increasing contribution of various sources of microplastics to freshwater systems, it is crucial to employ innovative, highly effective, and sustainable mitigation measures to protect freshwater resources, especially given the current overpopulation growth and water shortage in most countries worldwide.

Concerns about the impact of plastic and microplastic contamination have boosted public awareness and responsive actions. Schools have adopted instructional activities on plastics, non-governmental organisations have launched campaigns, and certain corporations have pledged to minimise plastic usage (Messing 2021). As an international response to the aggravating problem of microplastics, the USA enacted the Microbead-Free Waters Act in 2015 to ban the addition of plastic microbeads in the manufacturing of personal care products (McDevitt et al. 2017). In addition, other countries, including the European Union countries, have recently started to phase out plastic microbeads from numerous products like cosmetics (Wu et al. 2017). Europe also called for the recycling of plastic materials in 2018 by embracing the so-called European Strategy for Plastics in a Circular Economy as well as implementing other initiatives to protect the environment, such as "Zero Plastics to Landfill" (Du et al., 2021).

On the level of the Far East countries, China advocated "Opinions on Further Strengthening the Control of Plastic Pollution" at the beginning of 2020 (Du et al. 2021). Therefore, it is unequivocal that most countries seek the phase-out of plastics and search for sustainable alternatives. At the fourth United Nations Environment Assembly in March 2019, Officials (ministers) of the environment from more than 150 nations pledged to substantially eliminate single-use plastic goods by 2030 (Xu et al. 2021a). This action came after a previous assembly agreement highlighting the necessity of long-term microplastic removal from the oceans. Additionally, governments agreed three years ago, in May 2019, to modify the Basel Convention by officially asking for the importing countries' consent for contaminated plastic trash (Agamuthu et al. 2019). Moreover, many countries worldwide are now adding taxes on plastics that cannot be recycled to limit the production of these plastic materials (Silva et al. 2020).

## Toxicological profiles of microplastic exposure 

Microplastics have been found to have adverse effects on the environment and living organisms, including humans. Numerous studies have investigated the toxic effects of microplastics, including both in vitro studies (Choi et al., 2021a; Chan et al. 2017; Stock et al. 2021; Han et al. 2020; Hwang et al. 2020) and in vivo studies, primarily in marine organisms (Jin et al. 2018; Akhbarizadeh et al. 2018; Oliviero et al. 2019; Mateos-Cárdenas et al. 2019) and a few on rodents (Devriese et al. 2017; Li et al. 2020b; Santana et al. 2018). Moreover, studies have investigated the accumulation of microplastics from human samples in a clinical setting, including stool, colectomy samples, human placenta, and meconium (Wibowo et al., 2021; Ibrahim et al. 2021a; Braun et al. 2021). In the absence of epidemiological data, various in vitro studies have utilised different types of human cells to evaluate the effects of microplastics on humans (Danopoulos et al. 2021). The types of human cells used include human lung epithelial cells (Dong et al. 2020), human adenocarcinoma cell line (Wang et al. 2020b), human dermal fibroblasts (Hwang et al. 2020), peripheral blood mononuclear cells (Hwang et al. 2020), with a total of ten different types of human cells being used.

One of the issues is whether exposure to microplastics may lead to crucial adverse effects on human health. Based on this concern, Danopoulos et al. (2021) evaluated the exposure using meta-regression analysis on secondary data from different in vitro studies using human cells. A total of 168 publications were screened, and only 24 full articles were assessed. Seventeen full articles were eligible for the rapid review, and only eight proceeded for quantitative meta-regression analysis. The findings of the toxic effects on human cells were grouped into the biological endpoint categories: cytotoxicity, immune response, oxidative stress, barrier attributes, and genotoxicity. Among five biological endpoints, four were confirmed to be the effects of microplastics on human cells. For instance, irregular shapes of microplastics had significant biological effects. The minimal dosages of 10 μg/mL (5–200 μm) and 20 μg/mL (0.4 μm) were found to cause cytotoxicity and immunological responses, respectively. The human adenocarcinoma cell line cells are strongly associated with microplastic effects on cell viability. Additionally, the concentration of microplastics (g/mL) and exposure time significantly influenced cytotoxicity and immune response (Danopoulos et al. 2021). These findings suggest that exposure to microplastics may adversely affect human health, and further research is needed to fully understand these effects' scope.

Aquatic mammals have been reported to ingest various polymers, including polyether-sulphone, nylon, cotton, polyester, polyethylene, polypropylene, and ethylene-propylene (Nelms et al. 2019; Meaza et al. 2021). Microplastics can also contribute to the bioaccumulation of pollutants in aquatic mammals due to their hydrophobic surface and larger surface area-to-volume ratio (Nabi et al. 2022; Wang et al. 2020b; Verla et al. 2019). Besides, in vivo studies using marine organisms have shown that microplastics have significant toxic effects on animals through different exposure routes, such as intravenous, subcutaneous, intraperitoneal, oral, and skin exposure. The effects of microplastic exposure can vary depending on the route of exposure, either direct or indirect. Du et al. (2020) state that direct exposure occurs when pollutants come into direct contact with an organism, typically causing short-term acute toxicity. Indirect exposure occurs when microplastics and pollutants integrate into the food web, causing chronic organ toxicity.

Furthermore, in vivo have investigated the effects of various microplastic sizes, concentrations, and exposure durations. Most studies on marine organisms have focused on acute exposure rather than chronic exposure, and microplastics with sizes less than 5 mm have been commonly used. These studies have shown that microplastics accumulate and distribute in the gastrointestinal tract, gills, and fish muscles. Ingestion of microplastics in marine animals has been linked to alterations in gastrointestinal tract physiology, immune system depression, oxidative stress, cytotoxicity, differential gene expression, and growth inhibition (Oliviero et al. 2019; Meaza et al., 2021; Kedzierski et al. 2018; Nabi et al. 2019; Amin et al. 2020; Ugwu et al. 2021). These findings are confirmed by Danopoulos et al. (2021), who reported on the biological endpoint caused by microplastics to different human cells. In addition, studies have shown that microplastics can cause harmful alterations in the gastrointestinal tract physiology of marine organisms, such as an imbalance of gut microbiota in adult zebrafish, splitting of enterocytes, and cracking of villi (Jin et al. 2018; Lei et al. 2018). Qiao et al. 2019 also proved that after 21-day exposure to microplastics, the zebrafish exhibited microbiota dysbiosis, which altered the normal metabolism process (Qiao et al. 2019).

In addition to the effects observed in fish and mammals, microplastics were also found to cause adverse effects on coral and sea urchins. Tang et al. (2018) showed that acute exposure to microplastics activated the stress response in Scleractinia coral *Pocillopora damicornis* while suppressing its immune system and detoxification processes through the c-Jun N-terminal kinases and extracellular signal-regulated kinases signalling pathways (Tang et al. 2018). Meanwhile, Oliviero et al. (2019) reported that exposure to microplastics led to reduced larval length and blocked larval development of sea urchins, with the magnitude of the effect depending on the dose of exposure. Furthermore, Qiao et al. (2019) observed that microplastics induced oxidative stress in zebrafish by elevating catalase and superoxide dismutase levels in intestinal tissues and altering glutathione levels (Qiao et al. 2019). Overall, these studies demonstrate microplastics' potential wide-ranging harmful effects on different marine organisms.

Amphipods were the primary target of studies on the harmful effects of microplastics against invertebrates in the maritime environment. Several studies have reported that microplastics cause growth inhibition and decrease the growth of invertebrates. For instance, Deng et al. (2017) proved that microplastics could inhibit the growth of *Skeletonema costatum,* and freshwater algae *Chlorella pyrenoidosa* and *Tetraselmis chuii* were also inhibited (Davarpanah and Guilhermino 2019). In addition, chronic microplastic exposure can promote reproductive toxicity in *Daphnia magna*, *Daphnia pulex*, and *Ceriodaphnia dubia* (Jaikumar et al. 2019). Furthermore, Mateos-Cárdenas et al. (2019) reported that microplastic exposure to amphipods for 24 and 48 h did not significantly affect their mortality and mobility.

Besides marine organisms, several in vivo studies have examined the effects of microplastics on different animals, such as nematodes, *Oligochaeta*, arthropods, earthworms and rodents. Lei et al. (2018) reported that the size of the microplastics used affected the effects of microplastics on nematodes. In particular, exposure to 1.0 µm polystyrene at a concentration of 1 mg L^−1^ significantly downregulated gene expression associated with damage to cholinergic and gamma-aminobutyric acid-ergic neurons in nematodes. Similarly, Deng et al. (2017) found that the tissue accumulation of microplastics in mice was influenced by the size of the microplastics tested, with a significantly higher accumulation of 5 µm polystyrene in the kidney and gut compared to 20 µm polystyrene. The study also revealed that microplastics affected neurotransmission in mice. On the other hand, Zhu et al. (2018) found that the effects of microplastics on *Oligochaeta* were mainly dependent on the exposure concentration.

## Current knowledge and awareness of microplastic pollution

Various interrelated environmental issues exist today, such as the association between microplastic pollution, climate change, and biodiversity loss (Garcia–Vazquez and Garcia-Ael 2021). Rachel Carson, a renowned pioneer in environmental sciences, speculated about these interconnections in her influential book "Silent Spring," published in 1962 (Carson 2015). The correlation can be easily justified due to the high production of greenhouse gases while manufacturing microplastic-based products that require fossil fuels. Consequently, when these products are used, their waste materials are released into the aquatic environment, causing harmful effects on all living organisms, including phytoplankton, zooplankton, and top consumers (De Sá et al. 2018). This results in the disturbance of the entire ecosystem and the loss of species and ecosystem diversity, which cannot be restored.

It is worth mentioning that the public’s comprehension of these environmental issues, their root causes, their negative impacts, and their mitigation measures is a key solution and a quintessential step in tackling and controlling all these issues. However, the lack of basic knowledge, ambiguous facts, and the absence of clear information about environmental issues, particularly microplastic pollution, thus hinders the mitigation process of these issues (Deng et al., 2020b). In addition, a prevalent misunderstanding among the general public, including the well-educated, about the distinction between plastics and microplastics and the difficulty in identifying certain microplastic-based products exacerbates the issue. This was highlighted in a study that explored the knowledge levels of people in Shanghai, China, through surveys and questionnaires (Deng et al. 2020b). To address this, several measures must be implemented, which will be extensively discussed in this section, to enhance public awareness of microplastic concerns and facilitate the development of effective solutions.

The first step in microplastic control is to ensure that all aspects of microplastic issues, including their various origins, types, effects, fates, and other related factors, are covered in school and university curricula. By introducing this topic early on, students and young people can become familiar with the issue as early as possible. This approach could be implemented by teaching and connecting the microplastics issue through different subjects, as recently demonstrated in high schools in the San Diego area in the USA (Schiffer et al. 2019). For instance, chemistry courses taught students to differentiate between different types of plastics based on their properties and structures. Environmental science courses covered how these materials degrade into microplastics when released into the environment, and marine science courses explored their negative impacts on aquatic organisms. Additionally, students learned to apply computational models and machine learning techniques to investigate and speculate about plastic materials' degradation pathways and fate.

Furthermore, students should be encouraged to participate in research projects and write scientific reports to develop a solid background and offer practical solutions for microplastic issues. The American Chemical Society recently introduced new guidelines to the plastics and polymer industry and innovative research techniques to bachelor’s students in the USA, providing a great example of such an approach (Wenzel et al. 2015). Overall, it cannot be overstated how critical it is to introduce microplastic issues in school and university curricula by covering multiple aspects and involving students in critical thinking to suggest solutions to tackle this challenging and growing issue of microplastics.

The media has raised public awareness of microplastics in many countries, including the UK. The British Broadcasting Corporation (BBC), for example, has produced several documentaries and television shows that present the issue of plastic pollution in a simple and easily understandable way, encouraging the public to avoid using single-use plastic items. Through these efforts, the media has helped educate people about the impact of microplastics on the environment and motivated them to reduce their use of plastics (Henderson and Green 2020). The media is responsible for providing information and guidelines to the public and helping the constitutional authorities, political parties, and policymakers make the right decisions and reach real solutions for many urging environmental issues (Hansen 2018). In addition, the internet, in its different social media platforms, has recently constituted a powerful source for providing general and meticulous scientific information about microplastics (Garcia–Vazquez and Garcia-Ael 2021). In this regard, a group of researchers from Spain has recently tried to investigate the public’s response to the detrimental effects of microplastics on the marine environment by analysing more than 140,000 tweets on Twitter (Otero et al. 2021). The authors considered such investigation a vital tool in identifying the main spots of microplastic pollution worldwide by analysing the exact locations and languages of the posted tweets. Thus, it is unequivocal that everyone should be cautious about using plastic and microplastic-based products, seek to reduce their reliance on them as much as possible, and look for other environmentally friendly alternatives like bioplastics.

Another approach is the public’s perception of consumerism. Excessive consumerism became common in most countries, owing to the industrial revolution that started in the eighteenth century and, more specifically, after experiencing significant economic development and prosperity after the Second World War (Khan et al. 2020). Consequently, people started to experience the luxurious lifestyle and give more value to buying and those who purchase more. Such a societal concept was one of the main reasons behind the substantial increase in the amount of produced waste materials, not just limited to microplastics but also extending to other sorts of wastes, such as food, drugs and cosmetics, clothes, electric devices like phones and computers (Tamazian et al. 2009). Although changing the public’s societal behaviours is not reckoned an easy task, it is highly required to restrain the vast amounts of released waste materials and help the governments control the exacerbating issue of microplastics.

It is worth noting that in many countries, governmental policies have effectively reduced plastic consumption. For example, some countries have implemented bans, taxes, or pricing on plastic carrier bags, encouraging the public to use reusable bags and significantly reducing plastic consumption. In China, the use of plastic bags decreased by 49% following the introduction of a plastic bag ban (He 2012), while Botswana saw a 50% reduction in plastic bag use after implementing a plastic bag tax (Dikgang and Visser 2012). Similarly, Denmark achieved a 66% reduction in plastic bag use after implementing a plastic bag tax (Dikgang et al. 2012), and Portugal saw a 74% reduction after introducing a plastic bag tax (Martinho et al. 2017). In Washington, the use of plastic bags decreased by 80% following the introduction of a plastic bag fee (Romer and Foley 2011), and the UK saw reductions of between 8 and 85% after implementing a plastic bag charge (Poortinga et al., 2016). These examples illustrate the significant impact that governmental policies can have on reducing plastic consumption and mitigating the issue of microplastics in the environment. The implementation of these policies was not without challenges, given the numerous benefits that plastic carrier bags offer, such as sturdiness, longevity, water resistance, and more. However, the encouraging results demonstrated the effectiveness of controlling the utilisation of plastics and microplastics by enforcing restrictions, fostering international cooperation among different nations, and, most importantly, enhancing public awareness.

## Biological specimens for the detection of microplastics

Exposure to microplastic mainly affects the cellular and molecular components of living organisms. Understanding the origin, circulation, and susceptibility of microplastics in humans is essential for maintaining good health. Due to their position at the apex of the food chain, several animals, including humans, have been found to have accumulated microplastics in their circulatory systems (Sikdokur et al. 2020). Water consumption and food contamination significantly contribute to human microplastic exposure (Danopoulos et al. 2020). Food contaminated with microplastics, particularly seafood, is the primary source of exposure route for humans (Toussaint et al. 2019). It is also possible that people might be exposed to microplastics via air ingestion or through skin contact. When breathed in or consumed, microplastics may produce local particle toxicity stimulating immunological responses (Enyoh et al. 2020). A growing body of research suggests that people are often exposed to various plastics, ranging from microbeads to large bottles. As the evidence of microplastic exposure and the toxicity effect is prominent, it is necessary to assess the presence of microplastics in the human body through biological samples such as faeces, sputum, and placenta.

The reported in vitro and in vivo studies do not fully assess the risk of adverse effects of microplastics on human health, with some studies being conducted in the clinical setting. Specifically, the clinical studies examined the accumulation of microplastics from different human biological samples. For example, Wibowo et al. (2021) collected stool samples from healthy participants from a fisherman community living in the coastal region of Kenjeran, Surabaya, and Indonesia. They found that 50% of the participants were positive for microplastics in their stool, with high-density polyethylene spotted as the most predominant contaminant. However, Ibrahim et al. (2021a) reported that 100% of the sample collected had microplastic in human colectomy specimens, in which nine subjects had colorectal cancer, and another two were healthy subjects.

Interestingly, the study considered the potential microplastic airborne contamination and preventive steps. In addition, researchers found that samples taken from the human placenta and foetal meconium contained polyethylene, polypropylene, polystyrene, and polyurethane (Braun et al. 2021a). The study's primary limitation was that microplastics were detected in the control sample, indicating the possibility of contamination in the samples. Due to the potential high risk of environmental contamination with microplastics, clinical investigations are constrained. Therefore, precautions must be taken in clinical research in the future to avoid environmental contamination. Future research is also required to confirm and further explore the harmful effects of microplastics on human health as well as the underlying mechanisms. In addition, evaluating risk factors that may affect human exposure to microplastics is also beneficial.

### Microplastics in faeces

Microplastics are widely present in food and water sources, making human consumption unavoidable or unknowing. In a preliminary study, researchers used mass spectrometric analysis to examine polyethylene terephthalate and polycarbonate microplastics in faecal samples obtained from infants and adults. Although the polycarbonate microplastic content was the same in both groups, the researchers suspect infants may be more exposed to microplastics due to their frequent use of items such as bottles, teethers, and toys (Zhang et al. 2019). Fifteen different types of microplastics were identified in the faecal samples, with polyethylene terephthalate and polyamide being the most frequently detected (Yan et al. 2022a). It is unclear whether microplastic consumption causes a health concern. In recent research, microplastic content in the faeces of patients with inflammatory bowel disease was greater than that of healthy persons. These studies also indicate a strong correlation between the severity of inflammatory bowel disease and faecal microplastics (Yan et al. 2022a).

Detecting multiple types of microplastics in human faecal samples suggests that these particles are inadvertently ingested from various sources (Schwabl et al. 2019). While numerous studies have reported finding microplastics in human faeces, there is currently no standardised method for extracting them from these samples. One of the main challenges in extracting microplastics from human faeces is distinguishing between organic and inorganic materials. Digestion techniques involving nitric acid (HNO_3_), hydrogen peroxide (H_2_O_2_), potassium hydroxide (KOH), sodium hydroxide (NaOH), and enzymes are commonly used to extract microplastics (Yan et al., 2020). Plastic particles may be damaged by powerful chemical reactions and high temperatures, which necessitates the use of necessitating gentler procedures. Yan et al. (2020) suggested using Fenton's reagents for sample identification, nitric acid, and ethyl alcohol to break down materials and ethyl alcohol to remove residues on microplastic surfaces. This could preserve various types of plastic polymers in human faeces. Proteins, lipids, bacteria, and other faecal compounds must be digested for a comprehensive sample (Zhang et al., 2021c).

### Microplastics in sputum, saliva, and bronchoalveolar lavage fluid 

The contamination of microplastics in the air may have resulted from various sources, such as microfibre leakage into the water cycle from washing garments. However, direct release from textiles might significantly contribute to microplastic pollution, with less attention (Napper and Thompson 2016; De Falco et al. 2020). Modest amounts of microplastics in the respiratory tract have triggered the release of reactive oxygen species, which may lead to alterations in lung cell metabolism, proliferation, and cohesiveness (Goodman et al. 2021). The research identified 21 kinds of microplastics in sputum samples, with polyurethane constituting the majority. This research suggests that inhalation is a potential entry point for microplastics (Huang et al., 2022a).

Comparatively, a study conducted in Iran showed that saliva might not be a great choice for investigating the presence of microplastics in the human body as it exhibited relatively lower content than samples taken from hair and skin (Abbasi and Turner 2021). Bronchoalveolar lavage fluid obtained by instilling and recovering a saline solution from one or more lung segments may provide useful information about alveoli and foreign materials in respiratory airways (Sartorelli et al., 2020). Fourier transform infrared spectroscopy and scanning electron microscopy-energy dispersive spectroscopy proved the presence of microplastics in human bronchoalveolar lavage fluid. This finding correlates with the link between microplastic content and possibly damaged and decreased lung function (Baeza-Martinez et al. 2022).

### Microplastics in blood and placenta

Blood is an ideal biological sample for testing the presence of plastics because it is directly obtained from the body and does not come into contact with any plastics. Leslie et al. (2022) established the bioavailability of plastic microparticles in the human bloodstream. They found four high polymers used in plastics, such as polyethylene terephthalate, polyethylene, polymers of styrene, and methyl methacrylate, in the blood of 22 healthy participants. The researchers used steel syringe needles and glass tubes to avoid contamination and evaluated for background levels of microplastics using blank samples. According to new research, scientists have discovered microplastics for the first time in the human placenta, raising concerns that the compounds may interfere with embryonic development. Raman microspectroscopy was used to evaluate six human placentas collected from women who agreed to have their pregnancies monitored for microplastics. The sample was processed in a confined and controlled environment to avoid cross-contamination, revealing the presence of 12 microplastic fragments (Ragusa et al., 2021a).

In a separate study using the placenta, researchers developed a new technique analysing multiple contaminations for their plastic components, and the results were compared to the placenta, meconium, and maternal faeces. The samples collected through caesarean and breech deliveries enabled greater management of potential plastic contamination. Using pre-cleaned metal containers to store biological samples promptly readied samples for shipment and analysing negative samples ensures minimal cross-contamination, thus increasing the reliability of the result (Braun et al., 2021b). Table 2 highlights the biological specimens for the detection of microplastics.

**Table 2:** Biological specimens for detection of microplastics. Microplastic contamination was found in biological specimens such as blood, sputum, meconium, faeces, saliva, bronchoalveolar lavage fluid, and placenta

Study participants	Locations	Technique of analysis	Polymer types	Reference
Three meconium, six infants, and ten adult faeces	New York	Mass spectrometry	Polyethylene terephthalate and polycarbonate	Zhang et al. (2019)
Faeces of patients with inflammatory bowel disease and healthy people	China	Raman spectroscopy	Polyethylene terephthalate and polyamide	Yan et al. (2022a)
Faeces of eight healthy volunteers aged 33 to 65 years	Europe and Asia	Fourier transform infrared spectroscopy	Polypropylene and polyethylene terephthalate	Schwabl et al. (2019)
Sputum of 22 patients suffering from different respiratory diseases	China	Fourier transform infrared spectroscopy	Polyurethane polyester, chlorinated polyethylene, and alkyd varnish	Huang et al. (2022a)
8000 samples of saliva from adult	Iran	Raman spectroscopy	Not detected	Abbasi and Turner (2021)
Bronchoalveolar lavage fluid from 44 adult patients undergoing a bronchoscopy	Europe	Fourier transform infrared spectroscopy	Microfibres (rayon/viscose polyester cellulose and cotton)	Baeza-Martinez et al. (2022)
Blood samples from 22 healthy volunteers	Netherlands	Fourier transform infrared spectroscopy	Polyethylene terephthalate, polyethylene, and polymers of styrene	Leslie et al. (2022)
Placenta from healthy women and have a vaginal delivery	Italy	Raman microspectroscopy	Polypropylene	Ragusa et al. (2021a)
Placental tissue and meconium specimens during two caesarean sections for breech deliveries	Austria	Fourier transform infrared spectroscopy	Polyethylene, polypropylene, polystyrene, and polyurethane	Braun et al. (2021b)

The widespread contamination of microplastics is a concerning issue.

## Detrimental effects of microplastics ingestion on human health

**Fig. 5:**
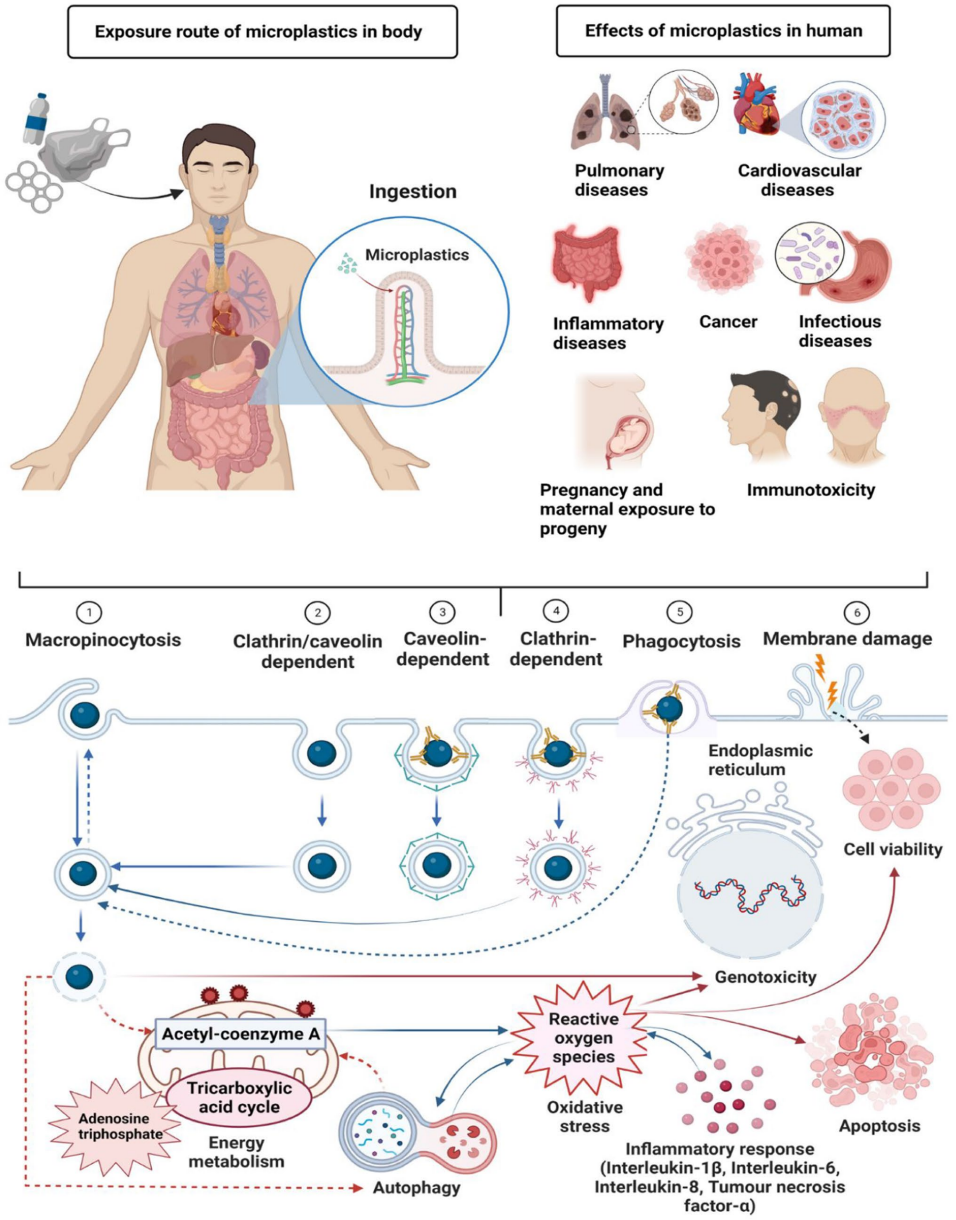
Detrimental effects of microplastic ingestion on human health and toxic mechanisms. Microplastics found in everyday items, including bottle packaging, can have harmful effects on human health when ingested. Once absorbed through the intestines, they can travel through the circulatory system to other organs. Different mechanisms can take microplastics, such as membrane damage, clathrin/caveolin-dependent, caveolin-dependent, clathrin-dependent, and micropinocytosis. High levels of microplastics can increase oxidative stress, producing inflammatory cytokines, apoptosis, cytotoxicity, and gene expression disturbances

The associated molecular mechanisms underlying microplastics' impacts on human health are summarised in Fig. 5. Exposure to the human body through ingestion of food containing plastic particles may pose potential health risks to humans, including cancer, immunotoxicity, intestinal diseases, pulmonary diseases, cardiovascular disease, inflammatory diseases, as well as pregnancy and maternal exposure to progeny. This section summarises the toxic mechanisms and effects of microplastics potentially causing harm to humans.

### Microplastic-induced cancer

Microplastics have recently been linked to several health problems, including toxicity and carcinogenicity, when consumed by humans (Gasperi et al. 2018; Blackburn and Green 2022). Due to the small size of microplastics, they have a high ratio of surface area to volume. Materials with a high surface area are highly cytotoxic to cells and tissue and can damage deoxyribonucleic acid** (**DNA) inside the cells. These mutations occur due to deoxyribonucleic acid damage that can lead to cancer (Campanale et al. 2020). Furthermore, uncontrolled waste of microplastics in water tends to absorb hydrophobic organic pollutants from water (Rodrigues et al. 2019). These harmful organic pollutants are carcinogenic, and long-term exposure can cause deoxyribonucleic acid mutations that contribute to cancer formation (Mishra and Rahi 2022). In addition, heavy metals such as arsenic (As), cadmium (Cd), chromium (Cr), mercury (Hg), and lead (Pb) used in the production of plastics are carcinogenic, according to the International Agency for Research on Cancer (IARC).

Epidemiological studies have shown that long-term exposure to microplastics is highly associated with cancer development in humans and animals (Karimi et al. 2021). Due to their small size, microplastics can be directly consumed by various marine organisms and contaminate the human food chain via the bioaccumulation process (Zhao 2022). Given the data and information on the levels of seafood consumed globally, humans are likely to be exposed to microplastics at a certain level (Campanale et al. 2020). For instance, a study by Smith et al. (2018) showed that the consumption of bivalves by Europeans was estimated to be up to 11,000 microplastics per person per year. Once consumed by humans, the particles of microplastics with sizes less than 2.5 mm can enter the digestive tract via a cellular process called endocytosis by the microfold cells of Peyer’s patches.

The amount of microplastic consumed influences the accumulative effect due to properties such as hydrophobicity and chemical composition. Based on the microplastic levels in humans at the gastrointestinal level, this hypothesis was further validated by microplastics in the human stool samples. These studies provide direct evidence of plastic consumption in humans that may lead to the development of various cancers (Campanale et al. 2020; Sharma et al., 2020).

Prata et al. (2020b) showed that microplastic intake might cause chronic inflammation and irritation, leading to deoxyribonucleic acid damage. Previously, it was also reported that the release of pro-inflammatory mediators that produce angiogenesis has resulted in the formation and progression of malignancies (Chang 2010a). For example, polycyclic aromatic hydrocarbons in food and water have become a general concern (Sharma and Chatterjee 2017). The cancer assessment risk study on the effects of microplastics evaluated by Sharma et al. (2020) revealed that microplastics adsorbed at around 236 ug/L of polycyclic aromatic hydrocarbons from water. This study demonstrates that microplastic leaching from plastic products is approximately 1000 times more dangerous than benzo[a]pyrene. The toxicological studies revealed that the toxic equivalent factor of microplastic linked with polycyclic aromatic hydrocarbons was calculated at 88.21 μg, implying that the cancer risk was 1.28 × 10^–5^ higher than the approved value of 10^6^.

Because microplastics are primarily absorbed through the stomach, they pose a risk of cancer development. Although several research works have explored the effects of plastic on humans, its impact on the stomach is still unclear (Campanale et al. 2020). Recently, Kim et al. (2022) demonstrated that long-term exposure to microplastics can increase the risk of stomach cancer. The presence of microplastics has caused the enhanced expression level of asialoglycoprotein receptor 2 (ASGR2). The elevated level of ASGR2 indicates the presence of typical cancer hallmarks such as CD44, N-cadherin, programmed death ligand 1 and proliferation. In addition, the excess exposure to microplastics caused a decrease in survival rate and an increase in the growth of tumours (Kim et al. 2022).

Wang et al. (2020c) reported that the different size of microplastics affects their toxicity in humans. The high toxicity to human cancer coli-2 cells (Caco-2) activity was observed in the microplastics with the size of 0.3 mm, 0.5 mm and 6 mm. Still, lower toxicity was demonstrated in 1 and 3 mm microplastic sizes. The toxicological studies were conducted by observing the reaction of Caco-2 cells to microplastics with different particle sizes. The uptake rates of microplastics were high (73%) at a small particle size and low (30%) at a large particle size. This finding shows that as the surface area is increased, the cellular oxidative stress is increased. Along with the adsorption characteristic, using bisphenol A as a plasticiser and microplastic nano-scale size has shown synergistic toxicity on Caco-2 cells.

On the other hand, a study showed that bisphenol A exposure could lead to local inflammation and affect colon cell permeability. This process was mediated by elevated levels of interferon-g, interleukin-17 and immunoglobulin A (Malaisé et al. 2018). Interferons are proteins that are part of human nature and defences. They signal the immune system when germs or cancer cells are detected in the body. Meanwhile, interleukin-17 promotes cancer cell survival and induces resistance to conventional chemotherapeutic agents (Bastid et al. 2020). In addition, an elevated level of serum immunoglobulin A has been observed in patients with breast, colon and liver cancers (Qiu et al., 2003).

Besides, exposure to bisphenol A caused similar effects on the colon cell and local inflammation in rats (Braniste et al. 2010). The process was related to the binding of bisphenol A to oestrogen receptor beta, primarily found in humans' intestines (Campbell-Thompson et al., 2001). The overexpressed colon cancer cells due to bisphenol A exposure in oestrogen receptor beta were associated with colon cancer growth. Bisphenol A appeared to block the oestrogen actions produced by the respective receptor. For example, the oestrogen-induced activation of the apoptotic cascade was impaired by the presence of bisphenol A, which affected the protection of endogenous oestrogen hormone in stopping colon cancer cell growth. Therefore, it can be concluded that bisphenol A exposure affects the immune functions and variation of microbiota, causing a pro-tumour inflammation in the human colon that favours colon cancer's growth (Bolli et al. 2010).

The digestive tract is another potential point of microplastic entry in humans. The studies conducted by Goodman et al. (2021) demonstrated evidence of microplastic in lung tissues with sizes smaller than 5.5 mm. The types of polymers used were polyethylene and polypropylene. Adverse health effects may be associated with the heterogeneous characteristic of these microplastics in the respiratory system.

Meanwhile, research studies by other groups have revealed that microplastics in human lungs affect cell proliferation and activate morphological changes (Amato-Lourenço et al. 2021). For example, different-sized microplastic was exposed to human alveolar A549 cells. It was further shown that sizes caused a significant reduction in cell proliferation with different cytotoxicity values. This disturbance at the proliferative levels of human cells proved that airborne microplastics might have a toxicological impact on cancer development (Amato-Lourenço et al. 2021).

The tri-o-cresyl phosphate, one of the isomers of plasticisers, is reported to have neurotoxic effects and cause liver and reproductive toxicity (Böckers et al., 2020). Investigations on the impact of tri-o-cresyl phosphate utilisation in microplastic showed that growth impairments affect reproduction and fertility in aquatic animals (Liu et al., 2020). Therefore, there is a high possibility of leaching from microplastics that affect the endocrine system. Another group (Böckers et al., 2020) studied the effects of tri-o-cresyl phosphate on human breast cancer cell line (MCF-7) and oestrogen receptor α human embryonic kidney-oestrogen receptors (HEK-ESR) cells. The study demonstrated that the coordination of tri-o-cresyl phosphate to oestrogen receptor α in silico had a high tendency to induce tumour growth by overexpressing angiogenesis and nutritional supply. This action promoted invasion and metastasis, affecting the cell cycle. Therefore, such action reveals that tri-o-cresyl phosphate exposure affects the endocrine system as oestrogen receptor α cells HEK-ESR and MCF-7 breast cancer cells.

Although exposure to plasticisers such as bisphenol A and tri-cresyl phosphate has been confirmed in the development of breast cancer, little is known about the mechanisms of cancer development. To obtain a clear view of the mechanism, Deng et al. (2021) exposed human breast cancer cell line (MCF-7) cells to bisphenol A at different concentrations and reaction times (Deng et al., 2021). This study showed that bisphenol A exposure significantly promoted the proliferation and migration of MCF-7 cells. Interestingly, the protein expression levels of pituitary tumour-transforming gene 1 (PTTG1) were enhanced considerably under bisphenol A exposure. Besides, the increased expression of PTTG1 was due to the inhibition of microRNA (miR-381-3p). The expression of miR-381-3p was low and exhibited an inverse correlation with the expression of PTTG1 in breast cancer tissues. Therefore, these findings reveal that bisphenol A can cause high protein expression of PTTG1 and affect the cell cycle to increase MCF-7 cell proliferation by suppressing the expression of miR-381-3p).

Similar to the pathophysiology of breast cancer, prostate cancer is also subjected to the activity of steroid and androgen receptors (Dobbs et al. 2019). It was demonstrated that the excess bisphenol A exposure could affect the deoxyribonucleic acid by breaking the double strand, causing instability of genomic and chromosome rearrangements. Moreover, the modifier effect of bisphenol A on the cellular epigenome and metabolome has the potential risk of causing secondary mutagenesis and tumour development (Allard and Colaiácovo 2010). Hu et al. (2021a) investigated the principal component analysis plot based on 96 trinucleotide context of sample prostate adenocarcinoma (PRAD-CA) and showed mutation spectra in the respective tumour sample. This result indicated that bisphenol A exposure leads to the damage of deoxyribonucleic acid and caused causes mutagenesis in human cells, thus inducing complex mutational effects in somatic genomes. Such findings are close to those in patients with stomach and ovarian cancer. Table 3 shows microplastics' impacts on cancer development and associated molecular mechanisms.

**Table 3:** Impacts of microplastics on cancer development and associated molecular mechanisms.

Biological effect/cancer type	Mechanism
• Chronic inflammation and irritation • Deoxyribonucleic acid damages	• Pro-inflammatory mediators • Progression of malignancies
• Lead to cancer hallmarks such as CD44, N-cadherin, programmed death ligand 1, and proliferation • Decreased survival rate • Increased the growth of tumours	• Enhanced the expression level of asialoglycoprotein receptors (ASGR2)
• Increased cellular oxidative stress	• The toxicological reaction of cancer-coli 2 (Caco-2) cells
• Inflammation and colon cell permeability are affected • Breast, colon, and liver cancers	• Elevated levels of interleukin-17 and immunoglobulin A • Induced resistance to conventional chemotherapeutic agents
• Cause liver and reproductive toxicity • Growth impairments • Breast cancer	• Overexpressing angiogenesis and nutritional supply • As oestrogen receptor α, the endocrine system mediates human embryonic kidney-oestrogen receptors (HEK-ESR) and human breast cancer cell line (MCF-7) breast cancer cells
• Breast cancer	• High protein expression of pituitary tumour-transforming gene 1 (PTTG1) • Increased MCF-7 cell proliferation by suppressing the expression of microRNA (miR-381-3p)
• Breast cancer • Prostate cancer • Secondary mutagenesis •Tumour development	• Breaking the deoxyribonucleic acid by double strands causes instability of genomic and chromosome rearrangements

Various cancer types can be developed due to microplastic exposure, which induces several inflammatory responses and deoxyribonucleic acid damage.

MCF-7 and RNA refer to the human breast cancer cell line and ribonucleic acid, respectively

### Immunotoxicity

Numerous immune cells underneath the intestinal epithelium coordinate the immune response by presenting antigens, generating antibodies, and releasing cytokines. Another element of the immunological barrier is secretory immunoglobulin A, mostly found on the surface of the human body's intestinal mucosa, which may interact with symbiotic bacteria to protect against infections (Shi et al. 2021; Hirt and Body-Malapel 2020a). In other words, the intestinal immune system defends against non-pathogenic commensal organisms and harmless food antigens while reacting quickly to infectious threats and toxins. Several mechanisms support this sensitive effort, including myeloid cells, innate lymphoid cells, and T cells. Immunotoxicity is the term used to describe the negative effects of pollutants on the immune system. Microplastics have been shown to have various immune system problems, such as immune cell death, altered surface receptor expression, and interleukin production (Sun et al. 2021).

The interactions between microplastics and the immune system may have immunotoxicity and adverse effects, including immunosuppression (decreased host resistance to infectious agents and tumours), immune activation (increased risk of developing allergic and autoimmune diseases), and abnormal inflammatory responses (chronic inflammation, tissue or organ damage and dysfunction) (Lusher et al. 2017). The absorption and toxicity of polymeric microparticles have been examined in mammalian systems (Wright and Kelly 2017; Blackburn and Green 2022). According to the research, microplastics affect the immune system and cell health. For instance, in rats, 10% of the dose was found in the gastrointestinal tract after a five-day oral course of 60 nm polystyrene nanoparticles (Hirt and Body-Malapel 2020a). Microplastics are not absorbed but remain attached to the apical region of intestinal epithelial cells. This action may result in intestinal inflammation and local immune system consequences. The primary location of microplastic absorption occurs in Peyer's patches with many microfold cells (Carr et al. 2012).

In another study, 0.3% of microplastics administered orally could penetrate the epithelium, demonstrating the ineffectiveness of microparticle excretion. The intestinal absorption of the particles may result in systemic exposure that is toxicologically significant. As a result, ingested microplastics can interact with intestinal tissues, enter the bloodstream, and probably stimulate the immune response (Bouwmeester et al. 2015). In this context, mice exposed to polyethylene microplastics (10–150 μm, 20 and 200 μg/g) for five weeks experienced changes in the serum levels of interleukin-1α and granulocyte colony-stimulating factor (G-CSF) (Li et al., 2020c). Additionally, the regulatory T cell count was lowered, and the fraction of T helper type 17 cells in splenocytes was increased. In a mice study of the cross-generational effects of polyethylene exposure (7 μm, 0.125 to 2 mg/day/mouse, for 90 days), blood neutrophil counts and immunoglobulin A levels were increased in the dams with spleen lymphocytes changed in both the dams and the offspring (Park et al. 2020).

Immunotoxicity caused by polycyclic aromatic hydrocarbons has been found in humans and animals. Numerous studies on human exposure have suggested that polycyclic aromatic hydrocarbons may stress the body's immune system. For instance, exposure to polycyclic aromatic hydrocarbons during pregnancy was significantly linked to higher percentages of a cluster of differentiation (CD), CD3^+^ and CD4^+^, lymphocytes and lower percentages of CD19^+^ and natural killer cells in umbilical cord blood. This finding suggests that exposure to polycyclic aromatic hydrocarbons during pregnancy may impact foetal immune development through changes in the lymphocyte distribution of the cord blood (Herr et al. 2010).

However, the molecular targets and mechanisms by which polycyclic aromatic hydrocarbons affect T lymphocytes’ immunotoxicity were not understood until the discovery of the global transcriptional activity of the B-activator protein in activated human T lymphocytes. B-activator protein inhibited chemokine ligand 12-induced T-cell chemotaxis, and trans-endosomal migration and interferon signalling pathways were activated (Liamin et al. 2018). For instance, concanavalin A-induced T cell proliferation in mice was considerably suppressed under B-activator protein exposure conditions, and the interferon, interleukin-2, and interleukin-4 were reduced (Guan et al. 2017). However, new research has identified several crucial immunomodulatory substances, including interleukin-27 and interleukin-28B, as immunotherapeutic agents for inflammation and lesions caused by polycyclic aromatic hydrocarbons (Majumder et al. 2020). Thus, ingestion of microplastics may affect the human body in various ways, such as altering intestinal homeostasis or altering immune cell recruitment or cytokine production levels. The vulnerability of the immune system to microplastics adds to the dangers to human health.

### Microplastic-induced intestinal diseases 

The intake of microplastics is around 39,000 to 52,000 particles per person per year (Cox et al. 2019). After inhalation, microplastic particles can enter the gastrointestinal system through food contaminated with microplastics or mucociliary clearance. This can lead to various negative health effects, including increased gut permeability, alterations in gut microbiome composition, and changes in metabolism (Salim et al. 2014).

Microplastics with a dimension greater than 150 μm are not absorbed. They remain bound to the intestinal mucosal layer and directly in contact with the apical part of the intestinal epithelial cells. This effect could lead to gut inflammation and a local impact on the immune system (Hirt and Body-Malapel 2020b). The smaller particles (dimension less than 150 μm) can cross the mucus barrier (Hirt and Body-Malapel 2020b). Several mechanisms of size-dependent uptake of nano- and microparticles have been explained, namely (i) endocytosis through enterocytes, (ii) transcytosis through microfold cells, (iii) crossing of the barrier by particles (persorption), and (iv) paracellular uptake (Powell et al. 2010). Although the intestinal uptake of microparticles is low (Carr et al. 2012), intestinal absorption of particles could lead to systemic toxicity as nanoplastics can infiltrate deep into organs (Hirt and Body-Malapel 2020b). Research has revealed that upon internalisation by human gastric adenocarcinoma cells, polystyrene particles can alter gene expression, reduce cell viability, and trigger pro-inflammatory responses and morphological changes (Forte et al. 2016).

The increasing prevalence of microplastics in consumer foods and beverages, the impact of plastics on the activity of the gut microbiome, and the potential for microplastics to degrade through digestion and interaction with intestinal microbes have been widely acknowledged (Tamargo et al. 2022a). Plastic particles found in foods have a major systemic and local negative impact on human health, such as mouth irritations or intestinal dysbiosis (Tamargo et al. 2022a). In addition, microplastic consumption may result in minor transcriptional alterations in the colon, indicating disturbances of the plasma membrane and mild inflammation (Rawle et al. 2022). The primary symptoms of microplastic intestinal toxicity are fatigue, diarrhoea, blood in stool, abdominal pain and cramping, reduced appetite, and unintended weight loss. These complications lead to cholera, gut dysbiosis, inflammatory bowel disease, irritable bowel disease, chronic bowel disease, metabolic disturbances, and other stomach issues.

Microplastic ingestion is more common in urban areas; however, an Indonesian study conducted in rural areas found microplastics in 7 of 11 collected stool samples. The concentration of microplastics found in the faeces was 6.94–16.55 μg/g (Wibowo et al. 2021). All colectomy samples collected from 11 adults in Northeastern Peninsular Malaysia contained microplastics in a study that used stereo- and Fourier-transformed infrared spectroscopy for analysis (Ibrahim et al. 2021b). This finding indicates that the prevalence of microplastics in the human gut system is becoming more prevalent and require more studies using human subjects. A study utilising a combined harmonised static model and dynamic gastrointestinal (SIMGI) model, which simulated various digestive tract regions in different physiological states, found that a single dose of polyethylene terephthalate microplastics undergoing biotransformations in the gastrointestinal tract and the colon, resulting in the production of different particles. Hence, microplastics can change human microbial colonic community composition, and the colonic microbiota could attach to the microplastics surface to induce biofilm formations (Tamargo et al. 2022b).

A systematic review of the effect of microplastics on the intestinal microbiota showed that they are potential triggers of intestinal dysbiosis, portrayed by the enrichment of *Chlamydia*, *Firmicutes*, and *Proteobacteria*. Exposure to microplastics resulted in increased intestinal permeability and the expression of immune signatures associated with inflammation, such as interleukin-6, interleukin-1α, interleukin-1β, tumour necrosis factor-α, and interferon -γ. This effect is likely due to microplastics trapping and stimulating intestinal inflammatory infiltration (Souza-Silva et al. 2022). Microplastics can also display structural changes in response to secondary exposure (Souza-Silva et al. 2022).

Microplastic analysis of faecal samples from healthy individuals and inflammatory bowel disease patients showed a significantly higher concentration of microplastics in patients with inflammatory bowel disease. In addition, 15 types were detected, and there was a positive correlation between faecal microplastics and inflammatory bowel disease status (Yan et al. 2022b).

An assessment was conducted on the impact of microplastics on lipid digestion in another study. The study demonstrated that five types of microplastics (i.e. polystyrene, polyethylene terephthalate, polyethylene, polyvinyl chloride, and poly(lactic-co-glycolic acid) significantly inhibited lipid digestion using an in vitro gastrointestinal system. Polystyrene showed the highest level of inhibition at 12.7%, and the study also found that lipid digestion decreased with increasing concentrations of polystyrene. The analysis suggested that microplastics reduced the bioavailability of lipid droplets by forming large lipid-microplastics heteroaggregates, adsorbing lipase, and altering the secondary structure of the enzyme. These findings indicate that microplastics can negatively impact lipid digestion, posing a human health risk (Tan et al. 2020).

A study evaluated the effect of polystyrene-microplastics consumption (0.5 μm size) for two weeks on mid-colon morphology. The study showed that microplastics reduced the thickness of mid-colon mucosa, muscle, flat luminal surface, and crypt layer. It was also noted that the microplastic treatment increased the expression levels of nucleotide-binding oligomerisation domain-like receptor pyrin domain-containing protein (NLRP) 3, apoptosis-associated speck-like protein containing a C-terminal caspase recruitment domain and cleaved caspase (Cas)-1 proteins. Additionally, the protein levels of inflammatory markers (i.e. nuclear factor kappa light chain enhancer of activated B cells (NF-κB), interleukin-6, tumour necrosis factor-α, interleukin-1β) were also increased in the treatment group (Choi et al., 2021b).

Consuming polyvinyl chloride microplastics at 100 mg/kg concentration for 60 days in adult mice reduced intestinal mucus secretion and enhanced intestinal permeability (Chen et al. 2022b). The treatment also reduced messenger ribonucleic acid expression levels of colonic mucus secretion-related genes, indicating a dysfunction in intestinal mucus secretion. This finding is supported by a reduced expression of messenger ribonucleic acid levels of genes related to colonic mucus secretion. Gut microbiota analysis showed that microplastic consumption changes the community composition of gut microbiota, for instance, lower *Verrucomicrobia* and *Epsilonbacteraeot* and higher *Firmicutes*, *Bacteroidetes*, *Tenericutes*, and *Patescibacteria* phylum abundance (Chen et al., 2022b).

A study investigated the effects of polyethylene microplastics on the progression of *Helicobacter pylori* infection. When mice were administered polyethylene microplastics or a combination of polyethylene microplastics and *Helicobacter pylori*, the results showed that they tested positive for *Helicobacter pylori* infection in the 10th and 14th weeks of the study. At the same time, those infected with *Helicobacter pylori* first and *Helicobacter pylori* alone were positive only in the 14th week after treatment (Tong et al. 2022). In addition, the microplastic fragments' diameter in the liver was greater than in gastric or intestinal tissues. In mice treated with a combination of microplastics and *Helicobacter pylori*, or microplastics followed by *Helicobacter pylori*, the rate of inflammatory cell infiltration was significant. The mice treated with a combination of microplastics and *Helicobacter pylori* showed the highest induction levels in the gastric organ index, myeloperoxidase, tumour necrosis factor-α, and interleukin-6. These findings suggest that the interaction between microplastics and *Helicobacter pylori* contributed to the improved colonisation of gastric mucosal epithelial cells, increased the efficiency of microplastics' entry into tissues, and induced gastric injury and inflammation in mice; thus, microplastics may provide a stable habitat for the growth of pathogenic bacteria such as *Helicobacter pylori* (Tong et al. 2022). Table 4 depicts the impacts of microplastics on the development of intestinal diseases and associated molecular mechanisms.

**Table 4:** Impacts of microplastics on the development of intestinal diseases and associated molecular mechanisms

Disease type	Biological effect	Mechanism	Reference
Gut dysbiosis	Human colonic microbiota changed Reduced *Staphylococcus* sp, *Bifidobacterium* spp., *Clostridium* spp., *Enterobacteriaceae* spp.	Colonic microbiota adherence to microplastics leads to biofilms formation	Tamargo et al. (2022b)
Gut dysbiosis	Colonic microbiota changes, inflammation	Enrichment of *Chlamydia*, *Firmicutes*, and *Proteobacteria*	Souza-Silva et al. (2022)
Gut barrier dysfunction and dysbiosis	Induction of gut barrier dysfunction and microbiota dysbiosis	Reduced intestinal mucus secretion Increased intestinal permeability Decreased expression messenger ribonucleic acid levels of colonic mucus secretion-related genes Modulation of gut microbiota composition	Chen et al. (2022b)
Gut inflammation	Transcriptional changes in the colon Inflammatory responses	Interaction between microplastics and the lumen side of the colonic epithelium Activating innate lymphoid cells, which could migrate to joint tissues and induce inflammation, worsens arthritis	Rawle et al. (2022)
Gut inflammation	Induced inflammation Increased nuclear factor kappa-light-chain-enhancer of activated B cells (NF-κB), interleukin-6, tumour necrosis factor-α, interleukin-1β protein levels	Decreased thickness of mid-colon mucosa, muscle, flat luminal surface, and crypt layer Increased nucleotide-binding domain, leucine-rich–containing family, pyrin domain–containing-3 (NLRP3). Apoptosis-associated speck-like, cleaved caspase-1 increases nuclear factor kappa-light-chain-enhancer of activated B cells (NF-κB ad p-IκB α) protein expression	Choi et al. (2021b)
Inflammatory bowel disease	Modulating the disease process in the induction of inflammatory bowel disease	Microplastic exposure is involved in disease progression Inflammatory bowel disease may enhance the retention of microplastics	Yan et al. (2022b)
Non-specific	Microplastics interact with lipid droplets and lipases, hence, reducing lipid digestion	Microplastics decreased the bioavailability of lipid droplets via the formation of large lipid-microplastics heteroaggregates Microplastics adsorbed lipase and changed the secondary structure of the enzyme	Tan et al. (2020)
Non-specific	Worsens *Helicobacter pylori* infection Inducing inflammation	Increased gastric organ index, myeloperoxidase, tumour necrosis factor-α and interleukin-6 Increased bacterial colonisation, improved microplastic entry into tissues, and promoted gastric injury and inflammation Microplastics act as stable habitats for bacteria	Tong et al. (2022)

Microplastic ingestion could cause gut dysbiosis by changing the colonic microbiota, inducing inflammation, causing gut barrier dysfunction, aggravating or inducing inflammatory bowel disease, affecting lipid digestion, and may worsening *Helicobacter pylori* infection.

### Microplastic-induced pulmonary diseases 

Microplastics have been detected in indoor and outdoor air; if inhaled, they could reach the human airway and lungs (Levermore et al. 2020). Previous studies have linked occupational exposure to airborne microplastics in workers of the synthetic textile, flock, and vinyl chloride or polyvinyl chloride industries to respiratory diseases such as airway and interstitial lung disease. In vivo studies have successfully replicated the lesions associated with these conditions (Prata et al., 2020b). Microplastics have been reported in human lung tissues obtained from autopsies (Amato-Lourenço et al. 2021).

Exposure to polystyrene nanospheres with a diameter of 64 nm has been shown to cause neutrophil influx and inflammation in rat lungs and proinflammatory gene expression in epithelial cells. This effect is most likely due to the high oxidant activity caused by the large surface area of the nanospheres. Additionally, exposure to microplastics has been shown to induce the expression of pro-inflammatory interleukin-8 protein in A549 epithelial cell lines (Brown et al. 2001).

In a separate study, researchers used natural lung surfactant obtained from porcine lungs to investigate the interaction between lung surfactant and microplastics (Shi et al., 2022b). The study demonstrated that microplastics altered the lung surfactant's phase behaviour, surface tension, and membrane structure. Interestingly, polystyrene adsorption of phospholipids components of lung surfactant was significantly higher than that of proteins. Polystyrene also expedited ascorbic acid and deoxyascorbic acid conversion, promoting hydrogen peroxide formation in the lung fluid containing surfactant and increasing hydroxyl radicals (Shi et al., 2022b).

A study found that polystyrene microplastics with diameters of 1–10 μm significantly inhibit the proliferation of human alveolar A549 cell lines. However, the microplastics had a little cytotoxic effect, as shown by trypan blue and Calcein-acetoxymethyl staining. Despite low cytotoxicity, further analysis showed a population-level decrease in metabolic activity parallel to the reduction in the proliferation rate. Additionally, microscopic examination revealed significant changes in cell morphology following exposure to microplastics. The uptake of 1-μm microplastics in cells can result in toxicological effects at the systemic level. (Goodman et al. 2021).

Xu et al. (2019) evaluated the effects of two different sizes of polystyrene nanoplastics (25 nm and 70 nm) on human lung A549 alveolar epithelial cells. They found that 25-nm polystyrene nanoplastics were more rapidly absorbed by A549 cells than 70 nm. The nanoplastics markedly decreased the cell viability, induced cell cycle deoxyribonucleic acid synthesis phase arrest, stimulated inflammatory gene transcriptions and modified the expression of proteins linked with cell cycle and pro-apoptosis. Nanoplastics also markedly induced upregulation of pro-inflammatory cytokines such as interleukin-8, nuclear factor kappa-light-chain-enhancer of activated B cells (NFκB), and tumour necrosis factor-α, as well as pro-apoptotic proteins (i.e. caspase 3, caspase 8, caspase 9, death receptor 5 and cytochrome c) (Xu et al., 2019). These results show that environmental nanoplastics could pose serious health effects on humans.

The same group also tested the effects of polystyrene nanoplastics on A549 cells and found that nanoplastics exposure increased migration and epithelial-to-mesenchymal transition markers, with the upregulation of reactive oxygen species and nicotinamide adenine dinucleotide phosphate (NADPH) oxidase 4 (NOX4). NADPH-NOX4 is a reactive oxygen species generator in the endoplasmic reticulum and mitochondria. Polystyrene nanoparticles also induced mitochondrial dysfunction, shown by membrane changes and declined cellular energy metabolism, and activated endoplasmic reticulum stress as demonstrated by the increased endoplasmic stress markers. Interestingly, NOX4 gene-silenced cells reversed these effects, which were confirmed by the involvement of NOX4 in epithelial-to-mesenchymal transition (EMT) induction in A549 cells (Halimu et al., 2022).

Exposure of polystyrene microplastics (1–1000 μg/cm^2^) to human non-tumorigenic lung epithelial cell line (BEAS-2B) caused pulmonary cytotoxicity and inflammation, with microplastics exposure above 1000 μg/cm^2^ inducing interleukin-6 and interleukin-8 production by inducing reactive oxygen species.

Microplastics can also impair the pulmonary barrier by reducing transepithelial electrical resistance by reducing zonula occludens proteins and the α1-antitrypsin levels in BEAS-2B cells. This finding shows that polystyrene microplastics inhalation can increase the risk of developing chronic obstructive pulmonary disease (Dong et al., 2020).

In a study, Sprague Dawley rats were exposed to 100-nm, 500-nm, 1-μm, and 2.5-μm polystyrene microplastics for three days. Intrathecal instillation of saline or 100 nm polystyrene with concentrations of 0, 0.5, 1, and 2 mg/200 μl was performed every two days for two weeks. The authors found that 100-nm and 1-μm polystyrene microplastics were deposited in the lungs, with alveolar destruction and bronchial epithelium disarrangement in the treated group. Pro-inflammatory cytokines, including interleukin-6, tumour necrosis factor-α, and interleukin-1β, were upregulated in the polystyrene microplastic group. Deoxyribonucleic acid sequencing showed upregulation of long non-coding ribonucleic acids (lncRNA XLOC_031479) and circular ribonucleic acids (circRNA 014,924 and 006,603, and downregulation of the expression of lncRNA XLOC_014188 and circ003982 in the treated group. These findings suggest that the identified circRNAs and lncRNAs may be essential in microplastic-induced lung inflammation (Fan et al., 2022).

Lu et al. (2021b) investigated the effects of microplastic exposure on normal and asthmatic physiology using a house dust mite-induced allergic asthmatic mouse model. Results showed that nasal microplastic exposure increased pulmonary inflammatory cells in normal mice and exacerbated airway inflammation in asthmatic mice. Immunofluorescent staining demonstrated increased macrophage accumulation and phagocytosis following microplastic exposure. Both normal and asthmatic mice exposed to microplastics exhibited increased mucus production and higher levels of immunoglobulin G1, whereas the microplastics plus asthmatic group showed significant effects on Immunoglobulin E. Moreover, microplastic exposure in asthmatic mice caused higher concentrations of interleukin-4, interleukin-5, and T helper 1 type tumour necrosis factor-α in bronchoalveolar lavage fluid. Bioinformatics analysis revealed that microplastics stimulated tumour necrosis factor and immunoglobulin production, activating a group of transmembrane B-cell antigens, cellular stress responses, and programmed cell death (Lu et al., 2021b). The impact of microplastics on the development of developing pulmonary diseases and associated molecular mechanisms is summarised in Table 5.

**Table 5:** Impacts of microplastics on the development of pulmonary diseases and associated molecular mechanisms. Microplastics can induce various harmful effects on lung health, such as inflammation, disruption of lung surfactant integrity, antiproliferative activity against human alveolar cells, lung fibrosis, loss of elasticity, exacerbation of asthma, and pathological changes that may lead to chronic obstructive pulmonary disease

Biological effect on the lung	Mechanism	Reference
Inflammatory responses	More significant neutrophil influx into rat lung after instillation of 64 nm polystyrene Increased lactate dehydrogenase and protein in bronchoalveolar lavage Increased expression of interleukin-8 in adenocarcinoma human alveolar basal epithelial (A549) cells	Brown et al. (2001)
Altering lung surfactant properties	Microplastics modified the phase behaviour, surface tension, and membrane structure of the lung surfactant Microplastic adsorbs phospholipid components of lung surfactants better and promotes the production of free radicals	Shi et al. (2022b)
Inhibition of human alveolar cells proliferation Potential toxicity	Population-level decrease in metabolic activity parallel to the reduction in the proliferation rate Significant changes in the morphology of cells exposed to microplastics of 1 μm	Goodman et al. (2021)
Reduced cell viability, induced cell cycle S phase arrest, stimulated inflammatory gene transcriptions and modified the expression of proteins linked with cell cycle and pro-apoptosis	Induced up-regulation of pro-inflammatory cytokines such as interleukin-8, NFκB and tumour necrosis factor-α, as well as pro-apoptotic proteins such as caspase 3, caspase 8, caspase 9, death receptor 5, and cytochrome c	Xu et al. (2019)
Increased migration and epithelial-to-mesenchymal transition markers Membrane potential changes and impaired cellular energy metabolism	Upregulation of reactive oxygen species and NADPH oxidase 4 (NOX4) Causes mitochondrial dysfunction Activation endoplasmic reticulum stress	Halimu et al. (2022)
Pulmonary cytotoxicity and inflammation by inducing reactive oxygen species in human non-tumorigenic lung epithelial cell line (BEAS-2B)	Increase expression of interleukin-8 and interleukin-6, and induce reactive oxygen species Disruption of lung epithelial barrier through oxidative stress and inflammation	Dong et al. (2020)
Induces inflammation, deposition of microplastics, lung histological changes	Alveolar destruction and bronchial epithelium disarrangement Interleukin-6, tumour necrosis factor-α and interleukin-1β were upregulated Modulation of lncRNAs and circRNAs	Fan et al. (2022)
Worsens airway inflammation Increased phagocytosis Increased cellular stress responses and programmed cell death in the asthma model	Increased pulmonary inflammatory cells Increased macrophages accumulation and phagocytosis Increased production of mucus, immunoglobulin G1, and Immunoglobulin E Increased interleukin-4, interleukin-5, and Th1 type tumour necrosis factor-α	Lu et al. (2021b)

NFκB is the nuclear factor kappa-light-chain-enhancer of activated B cells and NADPH is the nicotinamide adenine dinucleotide phosphate oxidase 4 (NOX4).

### Microplastic-induced cardiovascular diseases

The impact of microplastics on the cardiovascular system has garnered significant interest in both human and animal studies, given the potential for a range of health implications. Several studies have suggested that microplastics can have detrimental impacts on the cardiovascular system of humans. For instance, Lett et al. (2021) and Posnack (2021) highlight the effects of microplastics on human health, with a specific emphasis on the cardiovascular system and its potential to cause various health problems. The characteristics of microplastics, such as their size and chemical properties, strongly influence their interaction with human and animal systems, particularly in the cardiovascular system (Miller 2014).

Translocation is the process by which some microplastics can move through the digestive epithelium after entering the human body and be transported to the cells and other tissues by the circulatory system (Ribeiro et al. 2019). Microplastics internalise in humans via translocation, in which the particles pass through the intestinal epithelial cells or are absorbed by specific microfold cells (Prata et al., 2020b). In rats, microplastics with a size of approximately 0.90 mm entered the bloodstream within 15 min (Eyles et al. 1995). The particle size of microplastics influences the efficiency of translocation, and Paul-Pont et al. (2018) investigated various sizes of microplastics, which were less than 300 mm. Under normal circumstances, microplastics larger than 0.5 mm are difficult to move through the gastrointestinal wall. In normal circumstances, microplastics larger than 0.5 mm are difficult to move through the gastrointestinal wall (Lusher et al. 2020). Browne et al. (2008) showed that plastic particles smaller than 10 mm could move into the mussel's circulatory system and have more profound consequences.

Various studies have shown that microplastics exposure can cause cardiovascular toxicities in animals. Despite the complexity of understanding the mechanism that triggers the diseases mentioned above, recent studies have supported the idea that particulate matter causes oxidative stress, which results in cardiovascular damage, which can be similar to what effect would microplastic exposure produces on the cardiovascular system (Kelly and Fussell 2017). Pitt et al. (2018) found that exposure of zebrafish embryos to polystyrene microplastics resulted in the translocation of microplastic particles into the heart and a subsequent decrease in heart rate. Similarly, Wang et al. (2022) found that exposure of *Daphnia magna* to polyethylene microplastics of 20 and 30 mm size resulted in a suppressed heart rate. The study also revealed that different particle sizes had varying toxic effects on *Daphnia magna*, with larger microplastic sizes causing the degradation of amino acid metabolites.

Li et al. (2020b) investigated the effects of polystyrene on cardiac fibrosis in rats to understand better the mechanisms underlying how microplastics cause cardiovascular diseases. They found that microplastics triggered oxidative stress, leading to apoptosis in cardiomyocytes and the activation of the Wnt/beta-catenin pathway, resulting in cardiac fibrosis and dysfunction. Similarly, Wei et al. (2021) studied the impact of microplastics on cardiac tissues and discovered the role of pyroptosis and oxidative stress in cardiomyocyte injury. They found that microplastics exposure activated the nucleotide-binding oligomerisation domain-like receptor protein 3 inflammasomes in heart tissue, leading to inflammatory stimuli caused by oxidative stress that activated the Caspase-1-dependent signalling pathway. These findings shed light on the possible mechanisms by which microplastics cause cardiovascular diseases, although more research is needed in this area.

Zhang et al. (2022b) investigated the effects of microplastics on primary cardiomyocytes in chickens and proposed a mechanism for the observed effects. They found that microplastics disrupted antioxidant enzyme levels and increased levels of reactive oxygen species, leading to cardiac inflammation and pyroptosis. They suggested that the presence of microplastics altered several pathways, including nuclear factor kappa light chain enhancer of activated B cells-Nod-like receptor protein 3-gasdermin D (NF-κB-NLRP3-GSDMD) and adenosine monophosphate-activated protein kinase-peroxisome proliferator-activated receptor gamma coactivator-1α (AMPK-PGC-1α). This alteration produced oxidative stress, myocardial pyroptosis, inflammation, dysfunctional mitochondria, and energy metabolism (Zhang et al. 2022b).

Since humans can ingest microplastics through inhalation, exposure to airborne particles of microplastics may cause asthma, cardiac disease, allergies, and autoimmune diseases (Campanale et al. 2020). Recent research suggests that microplastics may adhere to the external membranes of red blood cells, potentially impeding their capacity to transport oxygen (Fleury and Baulin 2021). Lu et al. (2022) investigated the impact of polystyrene microplastics on human umbilical vein endothelial cells (HUVEC), revealing that microplastics with a size of 0.5 mm damaged the cell membrane and reduced mechanical stability. Meanwhile, smaller microplastics (about 0.1 mm) aggregated in the cytoplasm, damaging the cell membrane and disrupting autophagy. These findings provide new insight into the potential impact of microplastics on HUVEC and contribute to the health risk assessment of microplastics on the cardiovascular system. The stretching of red blood cell membranes caused by microplastics can reduce their mechanical stability, affect their ability to transport oxygen, and lead to symptoms such as shortness of breath, dizziness, and weakness (Lu et al. 2022).

Another situation concerning the effects of microplastic exposure on the human cardiovascular system is plasticiser additives such as bisphenol A and phthalate. These plasticisers are not covalently bound to the plastic matrix, so they are easily leached from plastic material (Campanale et al. 2020). Biomonitoring studies have raised concerns for the authorities as they have reported that 75–90% of the general population has detectable levels of these chemical additives (Ramadan et al. 2020). According to a 10-year cohort study by Bao et al. (2020), long-term exposure to bisphenol A was significantly associated with a hazard ratio of 46–49% for heart diseases. Furthermore, an epidemiological study has linked increased urinary phthalate and bisphenol A levels to a higher risk of hypertension, coronary artery disease, acute myocardial infarction, and reduced heart function (Ramadan et al. 2020). Data from a randomised controlled trial demonstrated that drinking water from a bisphenol A-containing bottle rapidly increased bisphenol A levels in urine, supporting the relationship between bisphenol A exposure and high blood pressure (Bae and Hong 2015).

On the other hand, phthalate can be regarded as a cardio-depressive agent. For instance, exposure to phthalate such as di(2-Ethylhexyl) phthalate (DEHP) can impact coronary circulation, leading to atrial contractile dysfunction. Furthermore, phthalate exposure may result in bradycardia, atrioventricular conduction disorder, and decreased cardiac conduction velocity (Jaimes III et al., 2019).

Epidemiological and population-based studies may find it challenging to determine the underlying cause of these diseases. The mechanisms that lead to these effects are likely influenced by various factors, including oxidative stress, hormones, and inflammation, as demonstrated in both population-based and experimental research (Posnack 2021). Therefore, more research is necessary to provide further insight into the effects of plastic chemical exposure on cardiovascular health.

### Microplastic-mediated infectious diseases

A study showed that the consumption of microplastics led to inflammatory changes in the colon and worsened viral arthritis. In mice consuming 80 μg/kg/day of microplastics dissolved in water, there was no apparent accumulation in major internal organs, lymphatic fluids, or intestinal tissues. However, the accumulation of microplastics led to significant transcriptional changes in the colon, potentially due to the interaction between microplastics and the lumen side of the colonic tissues, which could affect the mucosal epithelium and its barrier function (Rawle et al. 2022). Further research is needed to investigate the potential impact of microplastics on gastrointestinal health.

Consuming microplastics have been found to promote inflammation and prolong arthritic foot swelling in mice challenged with the chikungunya virus. This was associated with increased T helper type 1, natural killer cells, and neutrophil signatures (Rawle et al. 2022). The transmission of pathogens from ingested plastics to humans is still unclear and requires further research. The survival of these pathogenic organisms on plastic debris has not been thoroughly examined, and there is a need for more extensive studies to understand the transmission of pathogens and the associated risks of illness related to seafood consumption (Barboza et al. 2018).

### Microplastic-mediated inflammatory diseases 

Exposure to microplastics through contaminated food has been found to activate the immune system and decrease the number of gut microorganisms, potentially harming human health (Meaza et al., 2020). Studies have shown that microplastics can cause cellular toxicity in human immune and epidermal cells, as well as an increase in the production of inflammatory cytokines (Hwang et al. 2019). Chronic inflammation caused by microplastics can lead to oxidative stress and toxicity. Microplastics can exacerbate oxidative stress by being absorbed on the surface and producing reactive oxygen species during host inflammation episodes (Valavanidis et al. 2013). Larger microplastic particles have been shown to stimulate the production of various proinflammatory cytokines, including interleukin-6, interleukin-1b, and tumour necrosis factor-α (Green et al. 1998).

Researchers demonstrated that microplastics could interact with the surface of SARS-CoV 2 pseudovirus, increasing the infection rate. Inflammatory markers such as caspase 3, interleukin-8, and tumour necrosis factor-α genes may also influence the infection rate (Zhang 2022a, b and c). Caputi et al. (2022) demonstrated that microplastics increased inflammatory markers such as nuclear factor kappa-light-chain-enhancer of activated B cells (NF-κB), myeloid differentiation primary response 88 (MyD88), and pyrin domain–containing-3 (NLRP3) in terms of protein and gene expression in human gingival fibroblastic cells. Analysing the faecal sample of inflammatory bowel disease and healthy persons revealed a strong correlation between microplastic and the disease occurrence (Yan et al. 2022a). Inhalation of harmful plastic particles or their leachates seems to cause occupational diseases that result in an inflammatory response.

Inhalation of plastic particles may cause various lung reactions, including alveolitis, persistent pneumonia, inflammatory, and fibrotic modifications in the bronchial and peri-bronchial tissue and lesions in the interalveolar septa (pneumothorax) (Beckett 2000). Adducts and deoxyribonucleic acid mutations arise due to prolonged inflammation, leading to cancer formation. Inflammatory cytokines, oxidative stress, and immune system evasion may contribute to cancer formation (Chang 2010b). Higher cancer incidence is seen in synthetic textile workers with more than ten years of exposure and is linked with intensity, duration, and time since initial exposure (Acquavella et al. 1988).

### Pregnancy and maternal exposure to progeny or offspring 

There is rising worry over the damage that microplastics pose to human health. A healthy pregnancy depends on the complex regulation of the maternal-foetal immunological balance, but the risks of exposure to polystyrene in the first trimester are still unknown. The biological impacts and mechanisms of microplastic exposure during pregnancy are listed in Table 6.

**Table 6:** Impacts of microplastics on pregnancy and maternal exposure to progeny or offspring and associated molecular mechanisms

Biological effects during pregnancy	Mechanism	Reference
Alteration in the serum triglyceride, total cholesterol, high-density lipoprotein cholesterol, and low-density lipoprotein cholesterol levels in the mice's first filial offspring Alteration in the hepatic total cholesterol and triglyceride levels Changes in serum metabolites (amino acids and acyl-carnitines) between gender Changes of free carnitine (C0)/(palmitoylcarnitine, C16 C + stearoylcarnitine, C18) as an indicator of the potential risk of fatty acid metabolism disorder	Microplastic could affect the hepatic lipid metabolism Female and male offsprings react differently to maternal microplastic exposure during gestation (the specific mechanism is unknown) Peroxisome proliferator-activated receptors (PPARs) were key regulators of lipid and carbohydrate metabolism and in the modulation of inflammatory responses	Luo et al. (2019)
Polystyrene nanoplastics delivered to offspring increased brain and body weight of postnatal progeny Reduced the number of Kiel-67 + proliferative cells by more than 60%, lower progenitor cells positively labelled with nestin (a specific marker for neural stem cells) in the hippocampus Polystyrene nanoplastic exposure results in neurophysiological abnormalities and cognitive deficits in a gender-dependent manner	Acetylcholinesterase (AChE) inhibition and enhanced lipid oxidation (LPO) in the brain are two ways for microplastics to cause neurotoxicity Significant anomalies in brain development are caused by high doses of polystyrene nanoplastic (more than 500 g/day)	Jeong et al. (2022)
Reduced in number and diameter of uterine arterioles Reduced decidual natural killer cells percentage Increased helper T cells in the placenta Reverse M1 macrophage/M2 macrophage ratios Cytokine secretion shifts	The uterine blood flow is lessened because there are fewer and smaller uterine arterioles The macrophage subtype 1/subtype 2 ratio drastically changed to a dominant subtype 2 Cytokines switched to an immunosuppressive condition	Hu et al. (2021b)
Decreased birth and postnatal body weight Reduced liver weight Reduced testis weight, seminiferous epithelium, and sperm count Induced testicular oxidative injury	Microplastics either cause immunological and inflammatory responses or cell damage Unknown mechanisms contribute to the fertility rate declining over time	Huang et al. (2021b)
Reduced neurite length, the number of primary neurites, and the number of neurite branches Reduced the size of the hippocampal cell body Decreased neuronal viability and neuronal density in the hippocampus Impaired learning/memory Dysregulation of the expression of autism spectrum disorder-related genes in the hippocampus	Exposure of ospreys of both sexes to Bisphenol A caused longer neurites, more primary neurites, and more neurite branches but smaller hippocampus cell bodies. But bisphenol A exposure during pregnancy reduced the number of neurons and their viability in the hippocampus	Thongkorn et al. (2019)
Nano polystyrene deposition in the foetal liver, heart, kidney, and brain, as well as migration from the maternal lungs to the foetal compartment during exposure in late late-stage pregnancy	After exposure to nanoplastics through the mother's lungs, the foetal tissues may get affected There is conflicting evidence regarding how the blood–brain barrier develops and works in pregnancy. Thus, the blood–brain barrier may not have fully developed, leaving the foetal brain vulnerable to particle sedimentation	Fournier et al. (2020)

Maternal exposure to microplastics during pregnancy can negatively impact maternal and foetal health through various mechanisms, including inflammation and disruption of hormonal balance. Further research is needed to fully understand the extent of these effects and identify strategies to minimise exposure to microplastics during pregnancy.

According to Luo et al. (2019), metabolic abnormalities can be transferred to the offspring of pregnant mice exposed to 100 and 1000 μg/L of polystyrene at 0.5 and 5 μm. Additional research employing tandem mass spectrometry for various serum metabolites such as amino acids and acyl-carnitines revealed that 11 and 15 different metabolites changed significantly in the groups exposed to 0.5- and 5-μm microplastics, respectively. Most amino acids for the male first filial offspring tended to rise after maternal microplastic treatment. In contrast, most amino acids for the female first filial offspring tended to fall, demonstrating gender differences. Furthermore, the expressed hepatic genes confirmed the risk of fatty acid metabolism issues, as evidenced by alterations in free carnitine (C0)/(palmitoylcarnitine, C16 + stearoylcarnitine, C18), indications for clinical screening of hereditary illnesses. After maternal exposure to 5-mm microplastic therapy, the expression of genes involved in b-oxidation, such as peroxisome proliferator-activated receptor-alpha, acyl-coenzyme A oxidase, carnitine palmitoyltransferase, and medium-chain acyl-CoA dehydrogenase was inhibited, which may cause a problem with the body's energy supply.

In the offspring of pregnant and female nursing mice, Jeong et al. (2022) showed that maternal treatment of polystyrene nanoplastics during gestation and lactation affected the functioning of neural stem cells, neural cell compositions, and brain cell histology. The outcome demonstrated that maternally supplied polystyrene nanoplastics particles transferred to offspring led to increased brain and body weight of postnatal progeny at 10–500 μg/day doses, with an exaggerated effect at 500 μg/day. Exposure to high doses of polystyrene nanoplastics (500–1000 g/day) has been shown to significantly reduce the number of proliferating cells and progenitor cells positively labelled with nestin, which is a specific marker for neural stem cells. This reduction was more than 60% in the hippocampus, suggesting that polystyrene nanoplastics exposure impacts the functioning of neural stem cells in specific brain regions. As expected, exposure to polystyrene nanoplastics decreased neural stem cell proliferation, altered hippocampus neurogenesis, and visibly shorter neurite lengths in the neurons.

Further research is needed to investigate the potential link between elevated polystyrene nanoplastics exposure, gender, and an increased risk of neurodevelopmental abnormalities. In addition, gender appears to play a role in the effects of polystyrene nanoplastics on bidirectional synaptic plasticity, as studies have shown that the effects of exposure may vary qualitatively according to gender. Specifically, research has shown that the magnitude of long-term potentiation was significantly different in female mice exposed to polystyrene nanoplastics compared to controls. These female mice had 20% higher levels of gamma-aminobutyric acid in the hippocampus than male mice. These findings suggest that exposure to high levels of polystyrene nanoplastics may increase the risk of neurodevelopmental abnormalities and that this risk may differ based on gender.

According to Thongkorn et al. (2019), there are differences between the impact of prenatal bisphenol A exposure on genes associated with autism and their connections to sex-specific hippocampal functions. Ribonucleic acid-sequential analysis of hippocampus tissues demonstrated that prenatal exposure to bisphenol A altered hippocampal transcriptome profiles in a sex-dependent manner. Up to 5624 transcripts or 4525 genes were substantially differently expressed in hippocampi exposed to 5000 µg/kg maternal birth weight of bisphenol A rats compared to controls.

Hu et al. (2021a) investigated the effects of polystyrene nanoplastics exposure on the immune system of pregnant mice and their offspring. The results showed that exposure to polystyrene particles increased the resorption rate of embryos in mice, indicating potential toxicity to female reproduction. The percentage of CD45 + leukocytes and decidual natural killer cells in the peripheral blood, spleen, and placenta was significantly lower after polystyrene exposure, suggesting a drop in these immune cells. Additionally, the proportion of CD49b + natural killer cells in the CD45 + leukocytes significantly decreased throughout the first trimester, as they were the most prevalent immune cells in the placenta. The mononuclear subpopulations segregated from the peripheral blood, spleen, and placenta significantly differed between the two groups, indicating an impact on immune cell function. The pro/anti-inflammatory cytokines ratio was also affected by polystyrene exposure, with interleukin-4 increasing and tumour necrosis factor reducing. The study suggests that exposure to polystyrene nanoplastics during pregnancy can lead to immune system dysfunction and may increase the risk of adverse pregnancy outcomes.

Hu et al. (2021b) used flow cytometry to investigate immune system threats in an allogeneic mating murine model exposed to polystyrene particles. They found a significant increase in resorbed embryos in the microplastic-exposed group compared to the control group (16.31% versus 5.48%; p < 0.01), indicating potential toxicity to female reproduction. This is likely due to the absence of uterine arterioles, which are important for placental blood flow and protection against excessive oxidation and reactive oxygen species. CD45 + leukocytes and decidual natural killer cells significantly decreased after polystyrene exposure, with a notable drop in CD49b + natural killer cells during the first trimester. Mononuclear subpopulations from peripheral blood, spleen, and placenta significantly differed between the two groups. The pro/anti-inflammatory cytokine ratio was also affected by polystyrene exposure, with interleukin-4 increasing and tumour necrosis factor decreasing (*p *< 0.05). At the same time, interleukin-2 and interferon showed a modest decrease in messenger ribonucleic acid levels, and interleukin-6 tended to increase (Hu et al., 2021b). Huang et al. (2022b) found that maternal exposure to polystyrene nanoplastics during pregnancy and lactation in mice led to decreased birth and postnatal body weight in their offspring. In male offspring, high-dose exposure to polystyrene nanoplastics caused a reduction in liver weight, induced oxidative stress, inflammatory cell infiltration, increased proinflammatory cytokine production, and disrupted glycometabolism. Exposure to polystyrene nanoplastics during the pre-and postnatal period also reduced testicular weight, damaged the seminiferous epithelium, and reduced the number of sperm in mouse pups. Polystyrene nanoplastics were also found to promote testicular oxidative damage, indicated by increased malondialdehyde production and altered superoxide dismutase and catalase activity in the testis of mouse pups.

Fournier et al. (2020) conducted a study to examine the effects of maternal lung exposure to nano-polystyrene beads during late-stage pregnancy. On gestational day 19, pregnant Sprague Dawley rats were intratracheally injected with 2.64 × 10^14^ particles of 20-nm rhodamine-labelled nano-polystyrene beads. The study revealed that nano-polystyrene particles were found in the lungs, heart, and spleen of the mother, as well as in the placenta, foetal liver, lungs, heart, kidney, and brain, indicating translocation of nanoparticles from the mother's lungs to foetal tissues during late-stage pregnancy. Ragusa et al. (2021b) used Raman microspectroscopy to examine human placentas and found 12 microplastic pieces, 5 on the foetal side, 4 on the maternal side, and 3 in the chorioamnionitis membranes, indicating that microplastics can reach placental tissues at all levels once they enter the human body.

## Potential treatment strategies

The main focus of treatment strategies for microplastics is their removal from aquatic ecosystems, where they often end up. There are two broad categories of techniques for microplastic removal: conventional and innovative strategies. Conventional strategies include coagulation, membrane bioreactor technology, rapid sand filtration, and adsorption. Innovative techniques for microplastic removal include electrocoagulation, photocatalytic degradation, electrochemical oxidation, and magnetic separation. Each of these techniques has both positive and negative aspects, and the efficiency of microplastic removal is influenced by various factors such as the size and concentration of the microplastics, water flow rate, and pH. Table 7 summarises the different treatment techniques, reactions, and factors influencing their efficiency.

**Table 7:** Comparison of different treatment techniques used for the removal of microplastics

Treatment technique	Positive aspects	Negative aspects	Controlling factors	Reactions involved	Reference
Coagulation	Simple and fast operation, different coagulants can be used, remove various pollutants, relatively low cost	With a large volume of produced sludge, additives addition increase the cost and difficulty of dealing with different pollutants simultaneously	Type and dose of coagulant, pH level, pollutant charge, concentration	Charge neutralisation, adsorption, sweep flocculation	Xu et al. (2021b)
Membrane bioreactor technology	Removing different pollutants with various concentrations, high effluent quality, good removal efficiency	Aeration limitations, membrane fouling, the need to add nutritious materials to microorganisms, high cost	Pollutant load, membrane characteristics, flow rate, microorganisms	Combination of membrane filtration, including micro or ultrafiltration	Bayo et al. (2020)
Rapid sand filtration	Removing various pollutants, including viruses, small land area, low sensitivity to water quality parameters, high flow rate	Low efficiency, requires expensive flocculating materials, frequent maintenance, high cost	Flow rate, contact time, pollutant concentration	Flocculation, sand filtration	Bayo et al. (2020)
Adsorption	High removal efficiency, no sludge waste formation, various adsorbents could be used	Non-selective adsorption	Type and composition of adsorbent, coexisting pollutants	Electrostatic interactions, hydrogen bond interactions, π-π interactions	Zhang et al. (2021b)
Photocatalytic degradation	Eco-friendly, sustainable, high removal efficiency	High energy requirement (ultraviolet light)	Type and dose of photocatalyst, pH level, reaction temperature, pollutant concentration, light intensity	Electron transfer, formation of free radicals	Uheida et al. (2021)
Electrochemical oxidation	High efficiency, degradation of several organic pollutants, no need for adding chemical agents, no sludge formation	High cost of electrodes	Surface area and the material of the anode used, current intensity, type, the concentration of the electrolyte used, degradation reaction time	Anodic oxidation, indirect cathode oxidation	Chen et al. (2022a)
Electro-coagulation	No need for chemical coagulant materials, reduced operation time and cost, reduced amount of generated sludge, high efficiency with various water qualities	Need for frequent change of electrodes	Electrode efficiency, applied electricity, pollutant charge and concentration	Flocs formation, micro-coagulants formation, pollutant destabilisation	Kim and Park (2021)
Magnetic separation	High removal efficiency, various magnetic separators use to remove microplastics from sediment, freshwater, and seawater samples	Non-selective pollutant removal	Size and shape of the target pollutant	Electrostatic interaction, hydrogen bond formation, complexation	Shi et al. (2022a)

Conventional strategies for microplastic removal have been used for many years in water treatment plants and involve physical and chemical processes. In contrast, innovative microplastic removal techniques are still being developed and tested but hold promise for more efficient and effective removal of microplastics. It's important to note that while these techniques can be effective at removing microplastics from water, prevention is still the best solution. This includes reducing our use of plastic products and properly disposing of them to keep them out of the environment.

### Conventional treatment techniques

#### Coagulation

Coagulation is one of the most frequently utilised techniques for wastewater treatment. It uses various chemical agents (coagulants) to destabilise the dissolved and suspended particles and enables their removal by sedimentation (Shirasaki et al. 2016). Different coagulants, such as iron-based and aluminium-based coagulants, have varied removal pathways for microplastics. However, traditional methods of microplastic removal, such as charge neutralisation, adsorption, and sweep flocculation, remain relevant in describing their removal mechanisms (Zhou et al. 2021). Even though the coagulation process is one of the most common techniques used for wastewater treatment, it has several operational drawbacks, such as a high volume of resulting sludge that constitutes another environmental issue (Padmaja et al. 2020). This is problematic because the sludge generated from coagulation may contain more harmful substances than the original pollutants, leading to costly additional treatment and removal. Additionally, using additives to improve coagulation efficiency can increase the removal process's overall cost (Bahrodin et al. 2021).

The challenge of effectively treating multiple pollutants simultaneously has been identified as a major limitation of coagulation. The diversity in the composition of wastewater also contributes to the cost of the process, as various coagulants must be added, and extensive optimisation of reaction parameters is required to treat different types of contaminants (Natarajan et al. 2018). Due to these factors, the overall operational cost of the process could become too high to be feasible.

#### Membrane bioreactor technology 

Membrane bioreactor technology is a reliable method for treating municipal and industrial wastewater that usually contains various concentrations of different contaminants based on nitrifying bacteria and other microorganisms (Dvořák et al. 2013). Such a technology has been recently employed to remove microplastics from an actual wastewater treatment plant (Talvitie et al. 2017). The notable positive aspects of using membrane technology are high effluent quality and good removal efficiency with a high rejection potency towards target pollutants (Lares et al. 2018). However, certain issues still limit its removal efficacy, including aeration limitations, membrane fouling, and the need to add nutritious materials to microorganisms (Al-Asheh et al., 2021), which altogether may elevate the operation cost.

#### Rapid sand filtration

Rapid sand filtration removes different contaminants, such as viruses (Shirasaki et al. 2016) and suspended solids of clay particles (Nakazawa et al. 2021). This method has recently been acknowledged as a viable approach for removing microplastics from wastewater (Hidayaturrahman and Lee 2019). Rapid sand filtration has been identified as a promising method for microplastic removal due to its small land area requirement, low sensitivity to water quality parameters, and high flow rate (Talvitie et al. 2017). However, the effectiveness of this method is limited without the use of costly flocculating agents, and it requires frequent maintenance, which further adds to the overall cost of the filtration process (Enyoh et al., 2022).

#### Adsorption

The adsorption technique's superior efficacy in removing microplastics from wastewater has been proved by using various adsorbents, including chitin and graphene oxide (Sun et al. 2020a). In addition, other materials exhibited significant adsorption efficiency, achieving up to 100% for microplastics and even nanoplastics, such as layered double hydroxides (Tiwari et al. 2020). However, the non-selective characteristics of the adsorption pathway restrict the overall performance of this technique (Bruyninckx and Dusselier 2019). Therefore, future research efforts should prioritise enhancing the selectivity of adsorbent materials for microplastics to achieve better removal efficiency.

### Innovative treatment techniques

#### Photocatalytic degradation

The utilisation of photodegradation has been recognised as a highly effective and promising method for treating toxic organic pollutants, including microplastics, in wastewater (Liu et al. 2019). A semiconductor material absorbs visible or ultraviolet light in this process, generating free radicals, including reactive oxygen species such as singlet oxygen and superoxide radicals, which degrade the microplastics (Zhu et al. 2019). The photocatalytic semiconductor material absorbs light energy that exceeds its bandgap energy. It triggers an electron transfer from the valence band to the conduction band, creating positive holes in the valence band. This process ultimately generates superoxide and hydroxyl radicals, which break down the microplastics. The green synthesised iron-zinc oxide nanocomposite has recently emerged as a prominent semiconductor material Lam et al. (2021) used in the photocatalytic degradation of polyethylene. Despite its effectiveness, the photocatalytic method requires appropriate disposal of the residual sludge generated and careful monitoring to prevent any adverse effects on aquatic ecosystems (Lam et al., 2021).

#### Electrochemical oxidation 

Electrochemical oxidation is a sustainable and cost-effective technique for wastewater treatment that includes two methods, anodic oxidation and indirect cathode oxidation (Du et al. 2021). This technique has been shown to effectively degrade various organic pollutants, including microplastics, antibiotics, antipyretics, and dyes, into simple and non-toxic products such as carbon dioxide and water vapour without adding chemical agents (Du et al. 2021; Ouarda et al. 2018). Besides, electrochemical oxidation produces potent oxidants, such as hydroxyl radicals, hydrogen peroxide, and ozone, which efficiently degrade organic pollutants while avoiding the formation of any sludge waste (Kang et al. 2019). The electrochemical oxidation's efficiency is influenced by various factors, including the surface area and material of the anode, the current intensity, the type and concentration of the electrolyte used, and the duration of the degradation reaction (Kiendrebeogo et al. 2021). Therefore, this treatment technique is currently attracting significant attention from researchers.

#### Electrocoagulation

The electrocoagulation process is a prosperous, sustainable, and highly efficient technique for removing microplastics from wastewater, integrating the positive aspects of coagulation and electrochemistry (Moussa et al. 2017). Electrocoagulation produces flocs from the cations formed by metallic electrodes under an electric current. Subsequently, this process leads to the formation of "micro-coagulants" and the loss of suspended particle stability due to coagulation (Shen et al. 2020). Therefore, electrocoagulation is more efficient than conventional coagulation as it obviates the utilisation of chemical coagulant materials, consequently reducing the operation time and cost (Garcia-Segura et al. 2017). Moreover, electrocoagulation minimises the amount of sludge waste, produces water with lower total dissolved solids, and can be efficiently employed with different wastewater qualities. This sustainable and cost-effective method has gained the interest of researchers as an alternative to conventional coagulation methods.

#### Magnetic separation 

The efficiency of removing microplastics from wastewater using magnetic separation has been proven due to the lasting magnetic effect of the materials used and their high removal capacity (Zhang et al. 2021b; Abdel Maksoud et al. 2020). This technique has been recently applied in removing microplastics from sediment, freshwater, and marine water samples (Grbic et al. 2019). Various materials, known as magnetic seeds, are used in this removal process, including iron nanoparticles and magnetic carbon nanotubes. Magnetic separation can be regulated by electrostatic interaction, hydrogen bond formation, and complexation (Tang et al. 2021). However, the presence of other pollutants negatively affects the selectivity and the removal efficiency of microplastics (Jiang et al. 2020), while the size and shape of microplastics also affect the separation process (He et al. 2021). Consequently, more extensive research work is required to improve magnetic removal efficacy.

## Control strategies

Various strategies are available for controlling microplastics, which can be categorised as short-term and long-term. Each strategy has limitations, including high costs, as listed in Table 8. Therefore, selecting a particular strategy should consider factors such as a country's infrastructure, economic conditions, types of microplastics released, alternative options, and public readiness to transition to a non-plastic-dependent economy.

**Table 8:** Limitations of microplastic control strategies

Control strategy	Limitations
Reducing plastic and microplastic usage and production	It may not be feasible in some industries or for some products, could be expensive to implement, requires a shift in consumer behaviour, and may not address existing plastic waste
Behavioural changes towards plastic and microplastic products	Public fear of change, lack of trust in alternative products It may take a long time to be achieved, requires a shift in consumer behaviour, may not be feasible for everyone, and may not address existing plastic waste
Using biodegradable plastics	High production cost and low efficacy of bioplastics compared to conventional plastics. Not all biodegradable plastics are biodegradable, and they may not fully address the issue of plastic waste
Recycling and reuse of plastic waste	The unsuitability of recycling and reusing certain plastic wastes, such as medical wastes, particularly during the coronavirus disease-19 pandemic The process can be expensive and energy-intensive

Plastic control strategies are highly effective for managing plastic waste to mitigate plastic pollution and its impact on the environment and natural resources. However, some plastic materials, such as medical plastic waste, are more challenging to recycle, particularly in light of the coronavirus disease 2019 (COVID-19). Using biodegradable plastics or changing individual behaviours also has several challenges.

### Reducing plastic and microplastic usage and production 

One of the most effective strategies for controlling the release of conventional plastic and microplastic products into the environment is to reduce their utilisation and production (Peng et al. 2023; Yang et al. 2023). This is because prevention is generally better than treatment. An example of this strategy is the minimisation of microbeads in manufacturing personal care products and pharmaceuticals (Prata 2018). Although some critics argue that this approach only addresses one type of microplastic pollutant (Fältström and Anderberg 2020), it can still have a long-term impact in reducing the discharge of microplastic waste into water systems.

**Fig. 6:**
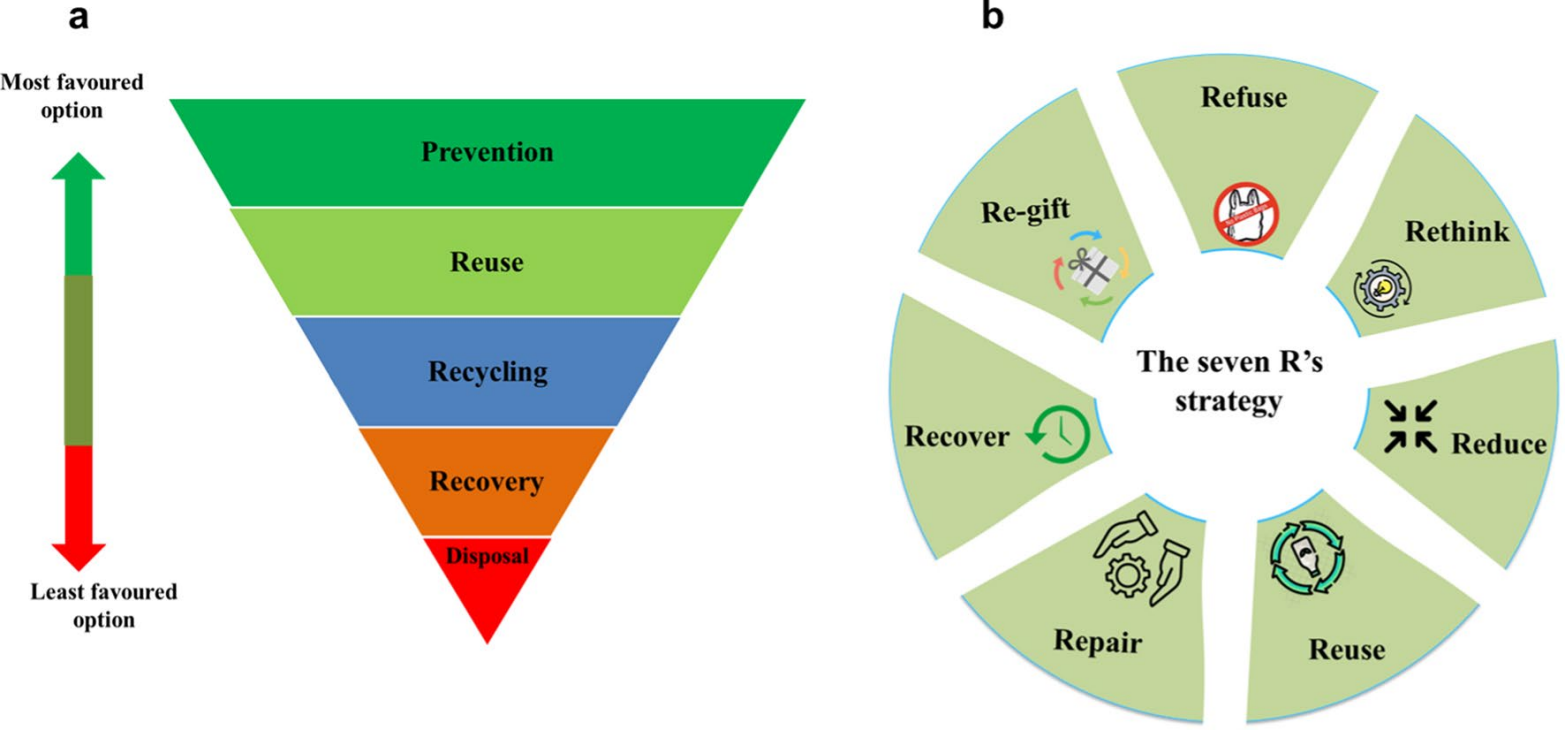
Plastic minimisation strategies. Strategies begin with prevention as the most favoured option **a**. Reuse, recycling, and recovery are other waste minimisation strategies. Disposal is the least favoured waste minimisation strategy. The 7 R’s waste minimisation approach includes recovering, repairing, reusing, reducing, re-gift, refusing, and rethinking **b**

The microplastic minimisation control approach follows an upside-down pyramid (Fig. 6a), starting with prevention, the most favoured option, followed by reducing, reusing, recycling, refusing, rethinking, regifting, recovering (7 R’s), and ending with disposal (Tsui and Wong 2019). Additionally, the 7 R’s strategy (Fig. 6b) offers different actions to minimise waste materials, including microplastics, from being released into the environment (Glavič, 2021). However, ruling institutions and individuals often overlook these options, particularly in developing countries, leading to massive amounts of microplastic waste (Azevedo et al. 2019).

Reusing and recycling plastic products is a highly effective strategy for managing plastic waste. While plastics used in packaging materials are relatively easy to recycle (Schyns and Shaver 2021), some plastic materials are more difficult to recycle, and there are public concerns about their use, such as medical plastic waste, particularly in light of the coronavirus disease 2019 (COVID-19) (P﻿rata et al. 2020a). Additionally, the increased use of single-use plastic products, such as face masks, during the pandemic has further complicated recycling efforts and exacerbated the issue of plastic waste (Silva et al., 2020). As a result, innovative solutions and increased efforts are needed to overcome these challenges and promote plastic recycling. Hydrothermal treatment also found not effective in plastic reusing, with only volume reduction can be obtained (Farghali et al. 2022a).

Subsequently, plastic minimisation is overlooked by most people, which is considered the main reason for creating and releasing massive loads of microplastic waste into the environment. To address this, media sources such as television shows, journals, and social media platforms have started to improve the general knowledge and awareness of microplastics in recent years. Implementing these waste minimisation strategies on a governmental and individual level is essential to effectively control microplastic pollution (Thiele and Hudson 2021).

### Behavioural changes towards plastic and microplastic products 

Encouraging changes in the everyday practices of individuals can have a significant impact on reducing the release of microplastics into waterways (Eagle et al. 2016). For example, individuals can opt for clothing made from natural fibres like cotton and wool instead of synthetic polymers such as polystyrene, acrylic, and nylon (De Falco et al. 2019). Installing a microplastic filter in washing machines can also help to reduce the amount of microplastic fibres released into the water (Gaylarde et al., 2021). Choosing natural materials in cosmetics and personal care products is another effective strategy to control microplastic pollution (Sun et al. 2020b). Additionally, avoiding single-use plastic items like bags, cups, and bottles and using alternatives made from glass materials can be a viable strategy (Tziourrou et al. 2021). However, implementing these behavioural changes can be challenging and requires a long-term effort.

### Using biodegradable plastics 

Biodegradable plastics, known as bioplastics, offer a promising solution for replacing conventional microplastics in various applications (Farghali et al. 2022b, Dhaka et al. 2022). These plastics have already been used in food and pharmaceutical packaging materials, such as polyhydroxyalkanoates, and in agriculture and horticulture as mulching films for soil and crop protection (Filiciotto and Rothenberg 2021; Zhang et al. 2020). Due to their lightweight and durability, bioplastics are also utilised in electric and electronic appliances, such as touch screens for smartphones and laptops, circuit boards, and data storage. They are also employed in the automotive industry to cover seats and airbags (Moshood et al. 2021). As a result, many potential applications for bioplastics with high efficacy exist.

## Conclusion 

Microplastics are a growing concern as a category of organic pollutants that have gained significant attention from researchers since 2014. As the impact of microplastics continues to increase, it is essential to develop sustainable solutions to mitigate their harmful effects and reduce their presence in the environment. This review examines various aspects of microplastics, including their types, shapes, sources, and global response. While microplastics can be found in multiple water bodies, land-based sources are the major contributors to environmental pollution (80–90%). The review also explores treatment techniques to mitigate their harmful effects, including conventional and innovative methods. In addition, we examined the toxic effects of microplastic exposure on human health, considering factors such as size, concentration, and exposure duration. The study has highlighted the relationship between the coronavirus disease 2019 (COVID-19) and the surge in single-use plastic item usage, particularly face masks, and explored different microplastic control strategies. To increase public awareness of microplastic concerns and promote the development of effective solutions, several measures must be implemented, including educational initiatives to raise individuals' awareness of microplastics and media sources like television shows, journals, and social media platforms. Various human biological specimens, such as faeces, sputum, saliva, blood, bronchoalveolar lavage fluid, placenta, and other organs, have been found to contain microplastics, suggesting that these particles may induce detrimental effects on human health. These effects can include potential health risks such as cancer, immunotoxicity, intestinal diseases, pulmonary diseases, cardiovascular disease, inflammatory diseases, and adverse effects on pregnancy and maternal exposure to progeny.

Several research gaps and issues require further examination and exploration in future studies related to microplastics. These include the need for more research on the impacts of microplastics on human health, identifying specific mechanisms underlying their harmful effects, exploring potential risk factors affecting human exposure, and developing effective mitigation strategies to promote public health. Further research is also needed to understand acute and chronic microplastic toxic effects on humans and animals and to develop suitable alternatives to single-use face masks and medical industry plastic waste. Microplastics must be converted into valuable by-products, improve their separation from other pollutants, and determine their environmental fate. Identifying suitable alternatives to single-use face masks is crucial while developing recycling and reuse methods for medical industry plastic waste. Furthermore, efforts should be made to improve the quality and efficiency of plastic alternatives, such as bioplastics, and to integrate microplastic treatment technologies to enhance their removal efficiency and minimise negative impacts. Finally, selecting a strategy to reduce plastic use should consider factors such as infrastructure, economic conditions, types of microplastics released, alternative options, and public readiness to transition to a non-plastic-dependent economy.
